# Cholesterol-dependent transcriptome remodeling reveals new insight into the contribution of cholesterol to *Mycobacterium tuberculosis* pathogenesis

**DOI:** 10.1038/s41598-021-91812-0

**Published:** 2021-06-11

**Authors:** Jakub Pawełczyk, Anna Brzostek, Alina Minias, Przemysław Płociński, Anna Rumijowska-Galewicz, Dominik Strapagiel, Jolanta Zakrzewska-Czerwińska, Jarosław Dziadek

**Affiliations:** 1 Laboratory of Genetics and Physiology of Mycobacterium, Institute of Medical Biology, Polish Academy of Sciences, Łódź, Poland; 2 Department of Immunology and Infectious Biology, Faculty of Biology and Environmental Protection, University of Łódz, Łódź, Poland; 3 Biobank Lab, Department of Molecular Biophysics, Faculty of Biology and Environmental Protection, University of Lodz, Łódź, Poland; 4 Department of Molecular Microbiology, Faculty of Biotechnology, University of Wrocław, Wrocław, Poland

**Keywords:** Microbial genetics, Pathogens, Microbiology, Bacteria, Bacterial pathogenesis

## Abstract

*Mycobacterium tuberculosis* (*Mtb*) is an obligate human pathogen that can adapt to the various nutrients available during its life cycle. However, in the nutritionally stringent environment of the macrophage phagolysosome, *Mtb* relies mainly on cholesterol. In previous studies, we demonstrated that *Mtb* can accumulate and utilize cholesterol as the sole carbon source. However, a growing body of evidence suggests that a lipid-rich environment may have a much broader impact on the pathogenesis of *Mtb* infection than previously thought. Therefore, we applied high-resolution transcriptome profiling and the construction of various mutants to explore in detail the global effect of cholesterol on the tubercle bacillus metabolism. The results allow re-establishing the complete list of genes potentially involved in cholesterol breakdown. Moreover, we identified the modulatory effect of vitamin B_12_ on *Mtb* transcriptome and the novel function of cobalamin in cholesterol metabolite dissipation which explains the probable role of B_12_ in *Mtb* virulence. Finally, we demonstrate that a key role of cholesterol in mycobacterial metabolism is not only providing carbon and energy but involves also a transcriptome remodeling program that helps in developing tolerance to the unfavorable host cell environment far before specific stress-inducing phagosomal signals occur.

## Introduction

Compared with other bacteria, *Mycobacterium tuberculosis* (*Mtb*) is capable of simultaneously consuming multiple carbon sources to augment growth^1^. However, numerous studies have established that in the nutritionally stringent environment of macrophage phagolysosome *Mtb* relies on fatty acids and cholesterol^2^. In our previous studies, we demonstrated that *Mtb* can accumulate and utilize cholesterol as the sole carbon source. Moreover, functional cholesterol degradation pathway is required for *Mtb* to multiply in human macrophages^3,4^. Other studies have demonstrated that *Mtb* employs a cholesterol‐dependent pathway to infect host cells, induces a foamy macrophage phenotype, and extracts cholesterol from caseous granuloma or serum-derived lipoproteins^5,6^. The ability to catabolize cholesterol is crucial during the early and late stages of *Mtb* infection^7–12^. Therefore, a thorough study on the cholesterol fate inside a mycobacterial cell is indispensable in understanding the tubercle bacilli survival success. Unfortunately, the process of cholesterol breakdown is complex, and an exceptionally large number of genes are annotated as involved in *Mtb* lipid metabolism^13^, thus greatly complicating the identification of genes involved specifically in cholesterol degradation. Therefore, robust categorization through transcriptomics may greatly help delineate genes tightly involved in this pathway. Through microarray-based transcriptional profiling, Nesbitt *et al*. identified over 200 *Mtb* genes that are regulated by cholesterol in a standard growth medium^9^. However, along with the emergence of RNA-Seq, an RNA sequencing technique that has an advantage over microarrays because it can produce perfectly quantified transcriptomes with a higher resolution, the growing body of evidence suggests that the lipid-rich environment may have a much broader impact on the *Mtb* transcriptome than previously thought. The use of RNA-Seq has allowed for the delineation of the transcriptional background of *Mtb* grown in even-length fatty acids^14^ or a mixture of lipids^15^. Although cholesterol is the key constituent of the *Mtb* lipid environment during pathogenesis, a complete transcriptomic landscape of the tubercle bacillus utilizing cholesterol as a sole carbon source is still lacking. Therefore, in this study, we applied RNA-Seq and constructed and analyzed specific mutants to precisely describe *Mtb* metabolic adaptations during growth on cholesterol as the only carbon and energy source and explain how cholesterol may facilitate the establishment of persistent infection.

## Results and discussion

### Differential gene expression of *Mycobacterium tuberculosis* H37Rv in the presence of cholesterol

**Figure 1:**
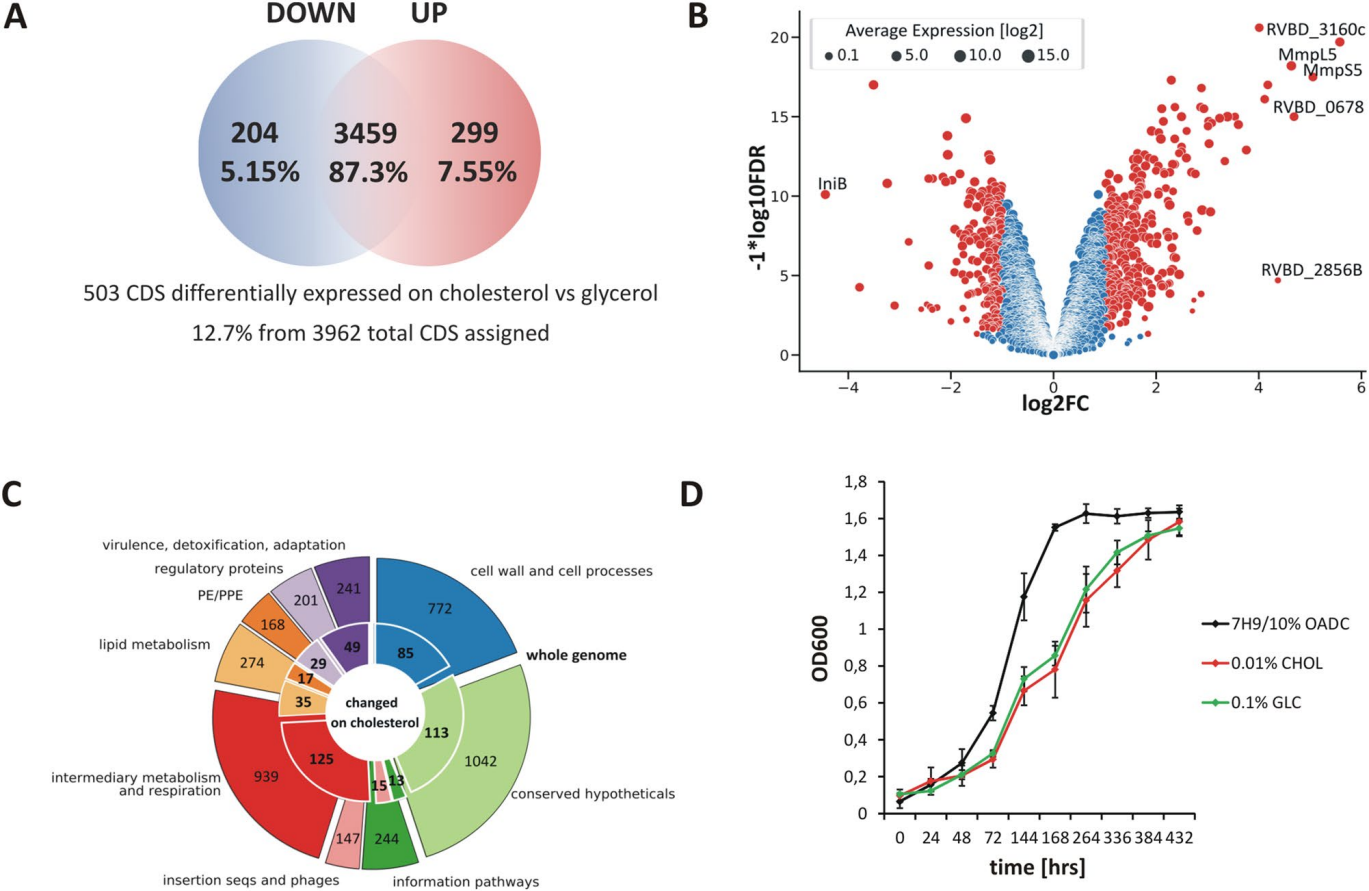
Summary of RNA sequencing results and growth characteristics of *M. tuberculosis* grown on cholesterol as the sole carbon source versus glycerol used as a control. (**A**) Venn diagram. A total of 503 differentially expressed CDSs were found. Among the 3962 CDSs assigned, 204 genes with decreased and 299 with increased expression were identified in the presence of cholesterol as the sole carbon source (**B**) Volcano plot. The negative log of false discovery rate (FDR) (base 10) is plotted on the Y-axis, and the log of the fold change (FC) (base 2) is plotted on the X-axis. The red points represent transcripts that are differentially expressed on cholesterol (FC > 1 and FDR < 0.05). The blue points represent transcripts that are not differentially expressed. The log average expression level (base 10) correlates with the size of individual points. (**C**) Donut plot. Protein functional categories were assigned to each transcript carrying information for protein synthesis. The number of genes with a change in expression observed for the individual categories (inner part of the chart) is plotted against the total number of transcripts encoding proteins belonging to the given category (outer part of the chart). Functional categories were assigned according to Mycobrowser (www.mycobrowser.epfl.ch, former Tuberculist). (**D**) Growth rate characteristics of the *Mtb* wild-type strain on 7H9/10% OADC medium and mineral medium supplemented with 0.01% cholesterol or 0.1% glycerol.

With a single change in providing cholesterol instead of glycerol as the sole carbon source the expression of more than 500 *M. tuberculosis* (*Mtb*) genes changed (Fig. 1A, Dataset S1). Genes demonstrating a change in expression with a false discovery rate (FDR) of <0.05 and a log_2_ fold change greater than an absolute value of 1 (changing two times or more) were considered differentially expressed (DE) in the study (Fig. 1B). Among the 3962 CDSs assigned, 204 genes with decreased (5.15%) and 299 with increased (7.55%) expression were identified (Fig. 1A). Interestingly, the vast majority of DE genes are not functionally related to lipid metabolism (Fig. 1C). The pool of DE genes was significantly enriched with operon members of more than 35 *Mtb* transcription factors (Dataset S2). We did not observe any difference in *Mtb* growth rate between the two main carbon sources discussed - glycerol and cholesterol (Fig. 1D), however, compared to the rich medium, *Mtb* demonstrates an extended Lag phase on both defined carbon sources which probably results from the need for complex metabolic adaptation (Fig. 1D). Conversely, the growth of propionate metabolism regulator (PrpR) mutant - ∆*prpR* that was used to study the transcriptional effect of methylcitrate cycle (MCC) inhibition is significantly retarded on cholesterol (Fig. 3A). Supplementation of the growth medium with the vitamin B_12_ that was used to analyze the influence of B_12_ on the *Mtb* transcriptome does not influence the bacterial growth rate (data not shown).

### Transcriptional profile of cholesterol uptake and degradation

Through microarray-based transcriptional profiling, Nesbitt *et al.* identified over 200 *Mtb* genes that may be regulated by cholesterol^9^. These genes include regulons of TetR family repressors KstR1 and KstR2^16,17^ situated in the Cho-region^9^ as well as genes that belong to the regulon of Mce3R^18^ or SigE^19^. However, cholesterol-induced changes were analyzed in a standard medium containing other carbon sources. Therefore, in our experiment, to determine the clear effect of cholesterol on the tubercle bacillus transcriptome, we applied a more accurate technique, RNA-Seq, to analyze the transcriptome of *Mtb* growing on cholesterol as the sole carbon source. The data were related to the results obtained on a minimal medium supplemented with glycerol. Additionally, the analysis was corrected for transcriptional change that is induced in standard 7H9/10% OADC medium.

**Figure 2:**
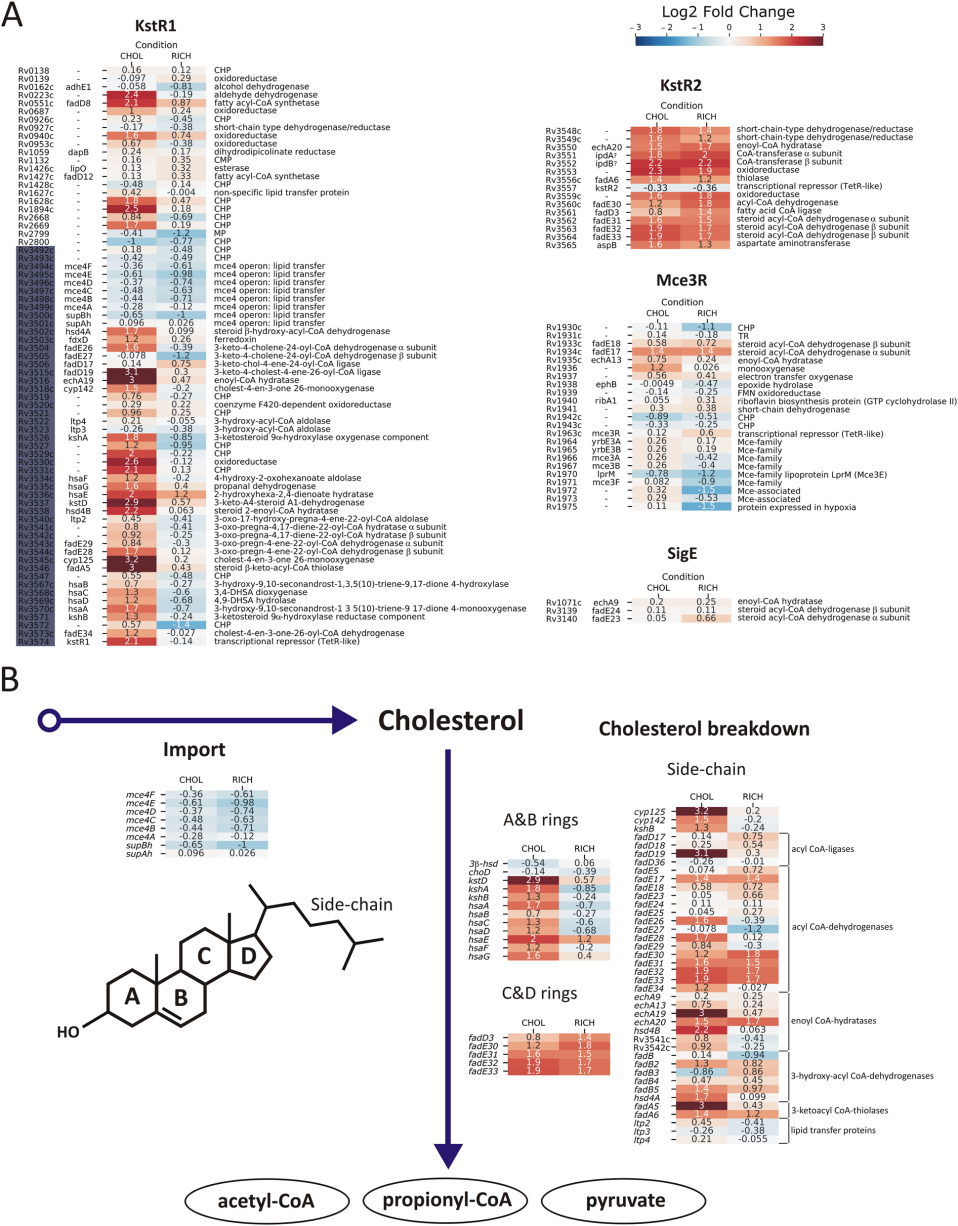
Differential gene expression of cholesterol import and breakdown genes. DGE analysis was performed comparing conditions of *Mtb* grown in glycerol vs cholesterol (CHOL) or glycerol vs 7H9/10% OADC medium (RICH). The results are presented as a heatmap of the log2-fold change in expression. See the color key for log2FC values. (**A**) Gene expression within KstR1, KstR2, MceR, and SigE regulon genes that were previously assigned as cholesterol-induced/associated. Genes that belong to the Cho-region^9^ are marked dark blue (**B**) DGE analysis of a functional compilation of genes that were previously classified in various studies^28^ as induced by cholesterol and involved in cholesterol import and breakdown.

The study confirmed the cholesterol-induced up-regulation of only 26 of the 83 Cho-region genes. Specifically, the results showed that only 32 of 71 KstR1 genes, including *kstR1* (*Rv3574*), were up-regulated, and the observed induction was strictly (excluding *hsaE*) cholesterol-specific (Fig. 2A, Dataset S1). Thirteen of 44 KstR1 genes previously classified as induced by cholesterol^9^ did not change their expression. Almost all KstR2 genes responded to cholesterol; however, the response was not specific because the same up-regulation was also observed in the 7H9/10% OADC medium. Therefore, the metabolite of A & B sterol ring degradation^20^ is surely not the only chemical inducer alleviating KstR2 repressor binding, and KstR2-regulated genes are probably also involved in β-oxidation of fatty acids. Despite being described as unique to cholesterol metabolism^21^ and involved in host-*Mtb* interactions^22,23^, the Mce3R regulon did not respond specifically to cholesterol, except *Rv1936* encoding putative monooxygenase. Similarly, none of the 16 genes of the SigE regulon changed its expression in our experiment.

**Figure 3:**
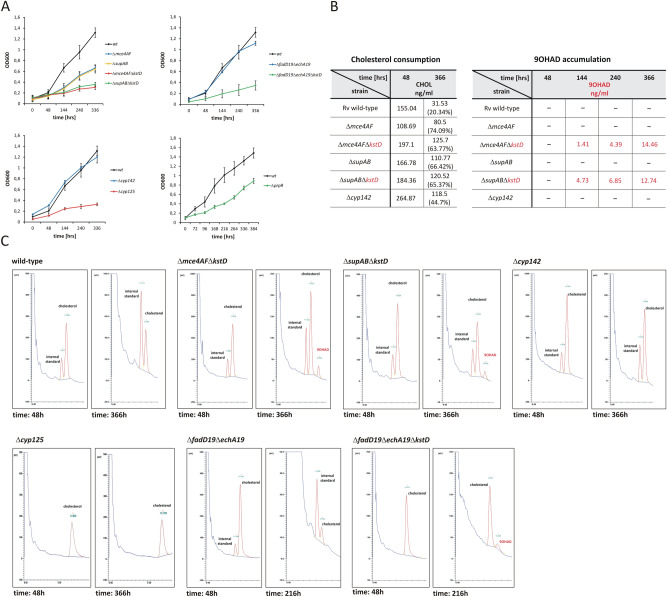
Phenotype of the *Mtb* mutants constructed in this study. (**A**) Growth rate analysis of the *Mtb* wild-type strain (wt) and *Mtb* mutants described in this study on mineral medium supplemented with 0.01% cholesterol. (**B**) Quantitative analysis of cholesterol bioconversion and 9-hydroxy-4-androstene-3,17-dione (9OHAD) accumulation by the *M. tuberculosis* wild-type strain and the *Mtb* mutants ∆*mce4AF*, ∆*mce4AF*∆*kstD*, ∆*supAB*, ∆*supAB*∆*kstD*, Δ*cyp142* growing in mineral medium supplemented with cholesterol as the sole source of carbon and energy, monitored by gas chromatography. Data concerning 9OHAD accumulation are marked red. The analysis was done in the selected time points. Equal amounts of the internal standard (4-androstene-3,11,17-trione) were added to each sample. The amounts of cholesterol and 9OHAD in the sample (nanograms per milliliter of the bacterial culture) were calculated from the ratio between the internal standard and the cholesterol and 9OHAD peaks. After adding cholesterol to the bacterial culture a 48-hour incubation was conducted for better cholesterol solubilization. Therefore, % of the initial amount of cholesterol in the sample was calculated considering the value measured at 48 hours as 100% (**C**) Chromatograms demonstrating cholesterol consumption and 9-hydroxy-4-androstene-3,17-dione (9OHAD) accumulation in *M. tuberculosis* wild-type strain and ∆*mce4AF*∆*kstD*, ∆*supAB*∆*kstD*, Δ*cyp142*, Δ*cyp125*, ∆*fadD19*∆*echA19*, ∆*fadD19*∆*echA19*∆*kstD* mutant cells. The selected time points are shown in the figure. Each time, the quantitative analysis was conducted, an equal amount of the internal standard (4-androstene-3,11,17-trione) was added to each sample.

Several studies have demonstrated that the *mce4* locus of the KstR1 regulon is involved in ATP-dependent cholesterol import^7,24,25^. Neither genes encoding putative permease *supA* (*yrbE4A*) and *supB* (*yrbE4B*) nor probable substrate-binding proteins (*mce4A*-*mce4F*)^26^ of *mce4* regulon were up-regulated by cholesterol in the described culture conditions (Fig. 2B, Fig. 5A). To date, prolonged hypoxia is the only reported condition that induces the whole *mce4* locus, suggesting its special role during persistence^27^. The essentiality of the permease component for the *in vitro Mtb* growth on cholesterol was previously determined^7^; however, the significance of the remaining genes of the *mce4* locus has never been assessed by *Mtb* knockout studies. Therefore, we constructed two *Mtb* mutants in which permease component genes (*supA*-*supB*) and accessory *mce4* genes (*mce4A*-*mce4F*) were deleted separately (Fig. S1A) to confirm their joint action in cholesterol transport. Both growth and cholesterol consumption were significantly impaired in the ∆*mce4AF* multiple mutant (Fig. 3A, B). However, 25% of the initial cholesterol content in the culture medium was still successfully metabolized (Fig. 3B). To confirm this observation, the ∆*mce4AF* mutant was subjected to deletion of the 3-ketosteroid Δ^1^-dehydrogenase gene (*kstD*) (Fig. S1B). During growth on cholesterol, the ∆*kstD* mutant accumulates the cholesterol degradation intermediate 9-hydroxy-4-androstene-3,17-dione (9OHAD)^3^. Therefore, the presence of 9OHAD in the lipid extract of ∆*mce4AF*∆*kstD* could confirm that cholesterol was successfully transported into the mutant cells. Chromatographic analysis revealed that after 144 hours of incubation on cholesterol the ∆*mce4AF*∆*kstD* strain starts accumulating 9OHAD (Fig. 3B, C), thereby confirming that the deletion of *mce4A*-*mce4F* only partially inhibits cholesterol transport. Similarly, analysis of the ∆*supAB* double mutant demonstrated that despite impaired growth it still metabolizes approximately 33% of the added cholesterol (Fig. 3A, B), which was then confirmed by the accumulation of 9OHAD in the ∆*supAB* strain with additional deletion in *kstD* gene - ∆*supAB*∆*kstD* (Fig. S1B, Fig. 3B, C). Our data demonstrate that all *mce4* genes are jointly required for optimal cholesterol transport; however, cholesterol does not influence their expression. Assuming SupA/B is the core cholesterol transporter, the incomplete inhibition of cholesterol transport in the ∆*supAB* mutant may strongly suggest the presence of another (less efficient in the described growth conditions) system of sterol uptake in tubercle bacilli.

Apparently, many genes within regulons previously described as regulated by cholesterol have shown no transcriptional response to this molecule. However, grouping them by functional categories revealed that almost every step of cholesterol breakdown was covered by significantly up-regulated genes (Fig. 2B). The expression of all ten genes required for sequential oxidation of the A & B ring carbon framework^28^ was cholesterol-induced in our study (Fig. 2B, Fig. 5A) and the induction was clearly cholesterol-specific. Prior to A & B ring degradation, cholesterol must be oxidized and isomerized to cholest-4-ene-3-one. The two genes encoding the enzymes able to catalyze this step - cholesterol oxidase ChoD and hydroxysteroid dehydrogenase 3β-HSD, are not cholesterol-regulated, and our previous study demonstrated that both are dispensable for cholesterol degradation in *Mtb*^29^. Since initial oxidation is an essential step of cholesterol breakdown, other proteins with this function must occur among mycobacterial dehydrogenases. Among the five genes that have been proposed to be involved in C & D ring metabolism^28^, four were up-regulated, although this change was not cholesterol-specific (Fig. 2B). In contrast to the findings of previous studies^9^, *fadD3,* encoding a probable acyl-CoA ligase introducing substrate for C & D ring metabolism, was not regulated by cholesterol.

The *Mtb* genome encodes 20 cytochrome P450 enzymes (Cyp)^13^ that have been proposed to be responsible for the initial step of cholesterol side-chain degradation. Cholesterol up-regulated four *cyp* genes (*cyp125*, *cyp136*, *cyp138* and *cyp142*), with the strongest increase observed for *cyp125* (Fig. 2B, Dataset S1). Cyp125, together with KshB reductase (also up-regulated), is responsible for the initiation of cholesterol side-chain β-oxidation *in vitro* however, is considered dispensable for cholesterol breakdown in *Mtb* H37Rv^30^ and Cyp142 is proposed as the functional backup for Cyp125^31^. Since *cyp142* was also moderately induced in our study, we decided to re-establish the involvement of both cytochromes in cholesterol degradation by the construction and analysis of ∆*cyp125* and ∆*cyp142* mutant (Fig. S1C). Surprisingly, our study demonstrates that along with its high level of cholesterol-dependent induction, *cyp125* is essential for *Mtb* growth on cholesterol. The ∆*cyp125* mutant did not grow on cholesterol (Fig. 3A) and was unable to metabolize it as a sole carbon source (Fig. 3C). Conversely, the deletion of *cyp142* did not disturb the growth on cholesterol (Fig. 3A). Cyp142 is, therefore, unable to provide compensatory activity for Cyp125; however, its cholesterol-dependent induction and slightly delayed cholesterol degradation in ∆*cyp142* mutant (Fig. 3B, C) may suggest unresolved cooperation of both proteins in cholesterol breakdown.

Among the 36 acyl-CoA ligase genes in *Mtb*, four (*fadD3*, *fadD17*, *fadD18*, and *fadD19*) were originally reported to be up-regulated on cholesterol^9^. In our study, only *fadD8* and *fadD19* showed cholesterol-dependent induction (Fig. 2A, B, Dataset S1). Both genes belong to the KstR1 regulon; however, only for FadD19 function in the first step of cholesterol side-chain thioesterification was demonstrated^32^. Within 34 genes of probable acyl CoA-dehydrogenases that may be involved in the first step of side-chain β-oxidation, only *fadE26*, *fadE28* and *fadE34* induction was cholesterol-specific (Fig. 2A, B).

Of the 21 genes annotated as *(S)*–specific enoyl–CoA hydratases involved in the second step of β-oxidation^13^, only *echA19* was specifically up-regulated on cholesterol (Fig. 2B, Dataset S1). In the *Mtb* genome, *echA19* is adjacent to the acyl-CoA ligase gene *fadD19*, which is also highly induced on cholesterol. Both genes are also up-regulated during infection of human macrophages or in artificial granuloma^33,34^. Therefore, EchA19 is considered the enzyme catalyzing hydration in the 1st cycle of cholesterol side-chain degradation, just after thioesterification by FadD19. However, our previous study on *M. smegmatis* demonstrated that FadD19 is required for the biodegradation of branched sterol side-chains but not straight ones, such as cholesterol, and EchA19 is not essential for the degradation of either substrate^35^. Since no experimental data were available in *Mtb*, we constructed an H37Rv mutant with a deletion within the *fadD19*/*echA19* locus (Fig. S1A). Strikingly, also in this species, phenotypic analysis of Δ*fadD19*Δ*echA19* double mutant demonstrated that both genes are dispensable for growth on cholesterol (Fig. 3A) and that the constructed strain consumes cholesterol as the wild-type *Mtb* (Fig. 3C). To further confirm undisturbed cholesterol side-chain degradation, the Δ*fadD19*Δ*echA19* strain was subjected to deletion of the abovementioned *kstD* gene (Fig. S1B). The presence of the 9OHAD intermediate in lipid samples of the Δ*fadD19*Δ*echA19*Δ*kstD* triple mutant growing on cholesterol finally demonstrated that cholesterol side-chain metabolism in *Mtb* devoid of *echA19* and *fadD19* genes was unaffected (Fig. 3C). These results indicate that despite significant induction by cholesterol, both genes may not be directly involved in cholesterol breakdown or other *Mtb* proteins within a large group of acyl-CoA ligases and enoyl-CoA hydratases may complement their essential enzymatic functions. *hsd4B* is the only cholesterol-induced gene annotated as coding for the probable *(R)*-enoyl-CoA hydratase that catalyzes hydration in the 2^nd^ round of side-chain β-oxidation (Fig. 2B).

Within three up-regulated β-hydroxy-acyl-CoA dehydrogenase genes (*fadB2*, *fadB5*, *hsd4A*), only *hsd4A* is required for growth on cholesterol, and it is located in the cholesterol regulon^36^. Among the 6 *Mtb* 3-ketoacyl-CoA thiolase genes, only *fadA5* was specifically up-regulated on cholesterol (Fig. 2B). Highly induced *fadA5* encodes thiolase of confirmed function, which is required for growth on cholesterol and up-regulated in macrophages and lungs^9,33,37^. The last carbon-carbon bond between the side-chain and the D ring is cleaved by aldolase, and the Ltp2 protein was demonstrated to conduct this step^38^. *ltp2*, together with two other homologous genes, *ltp3* and *ltp4*, was previously reported as cholesterol-regulated^9^; however, our study does not confirm this regulation (Fig. 2B).

### Flux of cholesterol catabolism metabolites: novel regulatory functions of PrpR (Rv1129) and vitamin B_12_ in mycobacterial virulence

**Figure 4:**
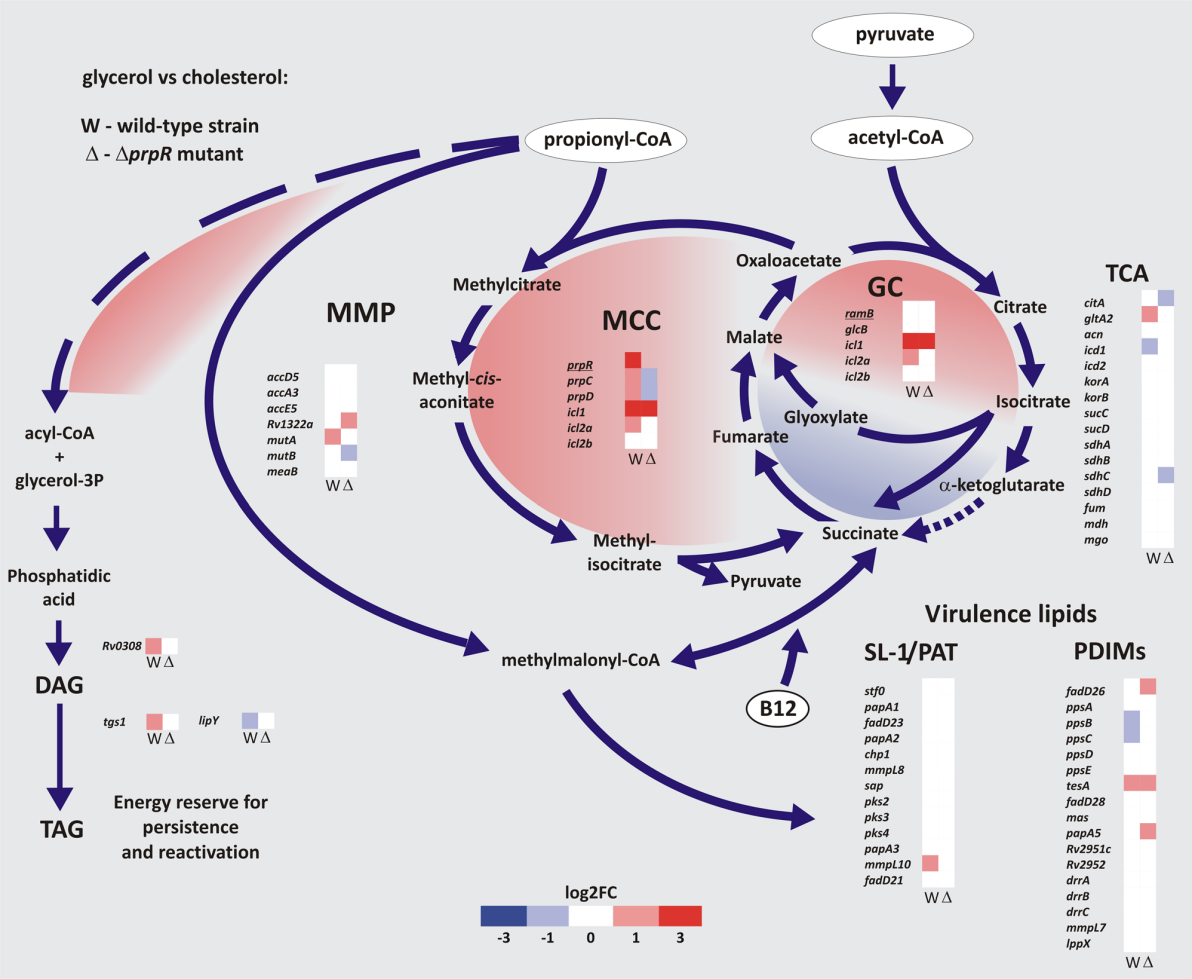
Transcriptional response of *Mtb* intermediary metabolism in channeling cholesterol degradation metabolites when cholesterol is utilized as a sole carbon source. DGE analysis of the main pathways of acetyl-CoA, propionyl-CoA, and pyruvate amelioration in *Mtb* and ∆*prpR* mutant. The two datasets represent DGE analysis comparing growth in glycerol vs cholesterol of the *Mtb* wild-type strain (W) and Δ*prpR* mutant (∆). Transcriptional changes that were not cholesterol-specific (were present also in standard 7H9/10% OADC medium) were excluded from the analysis. See the color key for log2FC values. *TCA* tricarboxylic acid cycle, *GC* glyoxylate cycle, *MCC* methylcitrate cycle, *MMP* methylmalonyl pathway. Up-regulated and down-regulated pathways are shown in red and blue, respectively.

**Figure 5:**
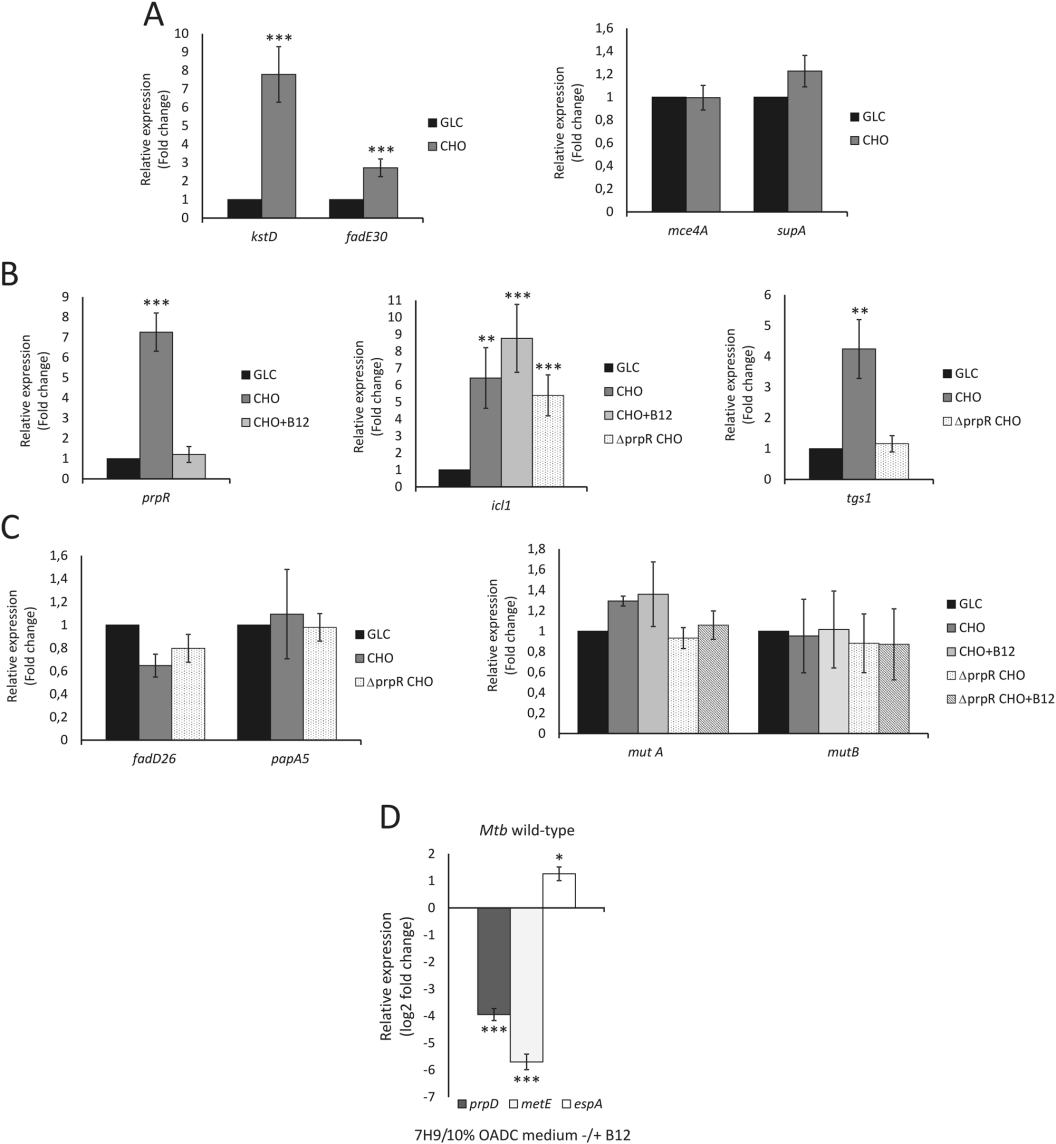
qRT-PCR validation of the expression of the selected genes in various growth conditions. Selected genes represent the main metabolic pathways discussed in this study. (**A**) Differential expression of the representative cholesterol breakdown (*kstD*, *fadE30*) and import (*mce4*, *supA*) genes. (**B**) Differential expression of the representative genes of *Mtb* central metabolism (*prpR*, *icl1*) and TAG synthesis gene *tgs-1*. (**C**) Differential expression of the representative genes of *Mtb* virulence lipid synthesis (*fadD26*, *papA5*) and the methylmalonyl pathway (MMP) (*mutA*, *mutB*). (**D**) Change in expression of PrpR operon genes (represented here by *prpD*) in response to vitamin B_12_ in *Mtb* wild-type growing in standard 7H9/10% OADC medium. The expression of *metE* was used as an example of confirmed B_12_-dependent regulation of gene expression. qRT-PCR analysis of *espA* (representative of the EspR regulon) expression in medium with or without B_12_ supplementation confirms the discussed influence of vit. B_12_ on the expression of ESX-1 associated genes. *GLC Mtb* growing on glycerol, *CHO Mtb* growing on cholesterol, *CHO+B12 Mtb* growing on cholesterol and vitamin B_12_, *∆prpR CHO* ∆*prpR* mutant growing on cholesterol, *∆prpR CHO+B12* ∆*prpR* mutant growing on cholesterol and vitamin B_12_. Statistical significance: one, two and three stars denote *p* values ≤0.05, ≤0.01 and ≤0.001, respectively (Student’s t-test).

Cholesterol consumption increases the cellular abundance of primary metabolites, mainly propionyl but also acetyl-CoA^39^. Due to the potential toxicity, their effective amelioration is a prerequisite for the ability to grow on this carbon source. We analyzed the transcriptional response of *Mtb* intermediary metabolism to delineate the contribution of different pathways in channeling cholesterol degradation metabolites (Fig. 4). Cholesterol utilization leads to substantial induction of the methylcitrate cycle (MCC), which is manifested by the massive increase in *prpR* (*Rv1129c*) propionate regulator gene expression along with *prpD*/*C* (*Rv1130*, *Rv1131*), which are directly controlled by this transcription factor (Figs. 4, 5B). We also observed induction of *gltA2* (*Rv0896*), an essential citrate synthase gene^36^ conducting the entry reaction of the tricarboxylic acid cycle (TCA), with concomitant repression of *icd1* (*Rv3339c*), which encodes isocitrate dehydrogenase, and strong induction of the isocitrate/methylisocitrate lyase (ICL/MCL) gene *icl1* (*Rv0467*). This finding confirms that in addition to the induction of MCC (propionyl-CoA disposal), *Mtb* cholesterol consumption induces the bypass of the oxidative part of TCA in favor of increased flux through the glyoxylate shunt (GS) for effective and carbon-saving acetyl-CoA disposal. The demonstrated changes clearly reflect those observed in the transcriptome of *M. bovis* BCG infecting human THP-1 cells^40^. Considering propionate the main metabolite of cholesterol breakdown, we decided to investigate the role of the key propionate disposal regulator PrpR in *Mtb* cholesterol metabolism by comparing the transcriptome of the *Mtb* wild-type and Δ*prpR* mutant growing on cholesterol (Fig. 4, Dataset S1, Dataset S2). Same as for the *Mtb* wild-type transcriptome, for DGE analysis the Δ*prpR* mutant RNA-Seq data were related to the results obtained on a minimal medium supplemented with glycerol and the analysis was corrected for transcriptional change that is induced in standard 7H9/10% OADC medium. PrpR deletion caused an obvious decrease in *prpC*/*D* expression, but the *icl1* level was still very high in the Δ*prpR* strain (Figs. 4, 5B). Although PrpR is a confirmed regulator of *icl1* during growth on propionate^41^, our results demonstrated that on cholesterol, which is not only a source of propionate but also acetate, *icl1* expression can be controlled jointly by regulators associated with MCC and/or GS. Conversely, we have shown that the second ICL/MCL gene, *icl2a* (*aceA*) (*Rv1915*), previously considered a pseudogene^42^, is also induced on cholesterol, but its up-regulation seems to be solely PrpR dependent (Fig. 4). Induction of *icl2a* was not observed on carbon sources whose metabolism does not generate propionate^14,42^. There are also no reports on the interaction between Icl2a and RamB, an acetate metabolism regulator, as was demonstrated for Icl1^43^. Icl2a may therefore be an enzyme associated mainly with propionate metabolism induced when the propionate level rises above a certain threshold, whereas Icl1 seems to be designated primarily to GS. Indeed, Icl1 possesses a 10-fold higher affinity to isocitrate than Icl2a^42^. We have also demonstrated that PrpR may control the cholesterol-induced TCA-GS transition through the regulation of *gltA2*, *citA,* and *icd1* expression (Fig. 4).

Functional MCC is required for *in vitro* growth of *Mtb* on cholesterol^44^ or in murine macrophages^45^. However, it has been found to be dispensable during infection in mice^45^, suggesting activation of other pathways of propionate disposal. During the *Mtb* lifecycle, carboxylated propionyl-CoA can also be incorporated into methyl-branched, cell wall virulence lipids (PDIM, SL-1/PAT)^46,47^. Importantly, this anabolic pathway not only ameliorates the toxic excess of propionate but also dissipates reducing equivalents that arise during lipid β-oxidation^45,48^. Our study did not show clear cholesterol-induced transcriptional changes within PDIM or SL-1/PAT metabolism (Figs. 4, 5C). It confirms that virulence lipid synthesis is largely posttranscriptionally regulated^48^ and depends simply on common precursor availability^47^. Interestingly, we have shown that inhibition of MCC during growth on cholesterol through *prpR* deletion increases the expression of the WhiB3 (Rv3416) redox sensor directly modulating virulence lipid synthesis in response to reductive stress^48^. This finding suggests an interplay between MCC and virulence lipid synthesis in cholesterol metabolite amelioration. Surprisingly, we also noticed a cholesterol-induced increase in the expression of the triacylglycerol (TAG) synthesis genes *tgs-1* (*Rv3130c*) and Rv0308 with concomitant down-regulation of the LipY lipase gene, which is involved in TAG hydrolysis upon nutrient starvation. The shift in *tgs-1* expression on cholesterol was not observed in the ∆*prpR* mutant (Figs. 4, 5B). Since *tgs-1* is a member of the DosR/S/T regulon, synthesis of TAG, which acts as an energy reserve for reactivation, has been strictly associated with entering dormancy to date^49,50^. However, it appears that in a cholesterol-rich environment, *Mtb* can initiate storage lipid synthesis without induction through DosR/S/T, even in aerated *in vitro* culture, and this process may be PrpR-dependent.

**Figure 6:**
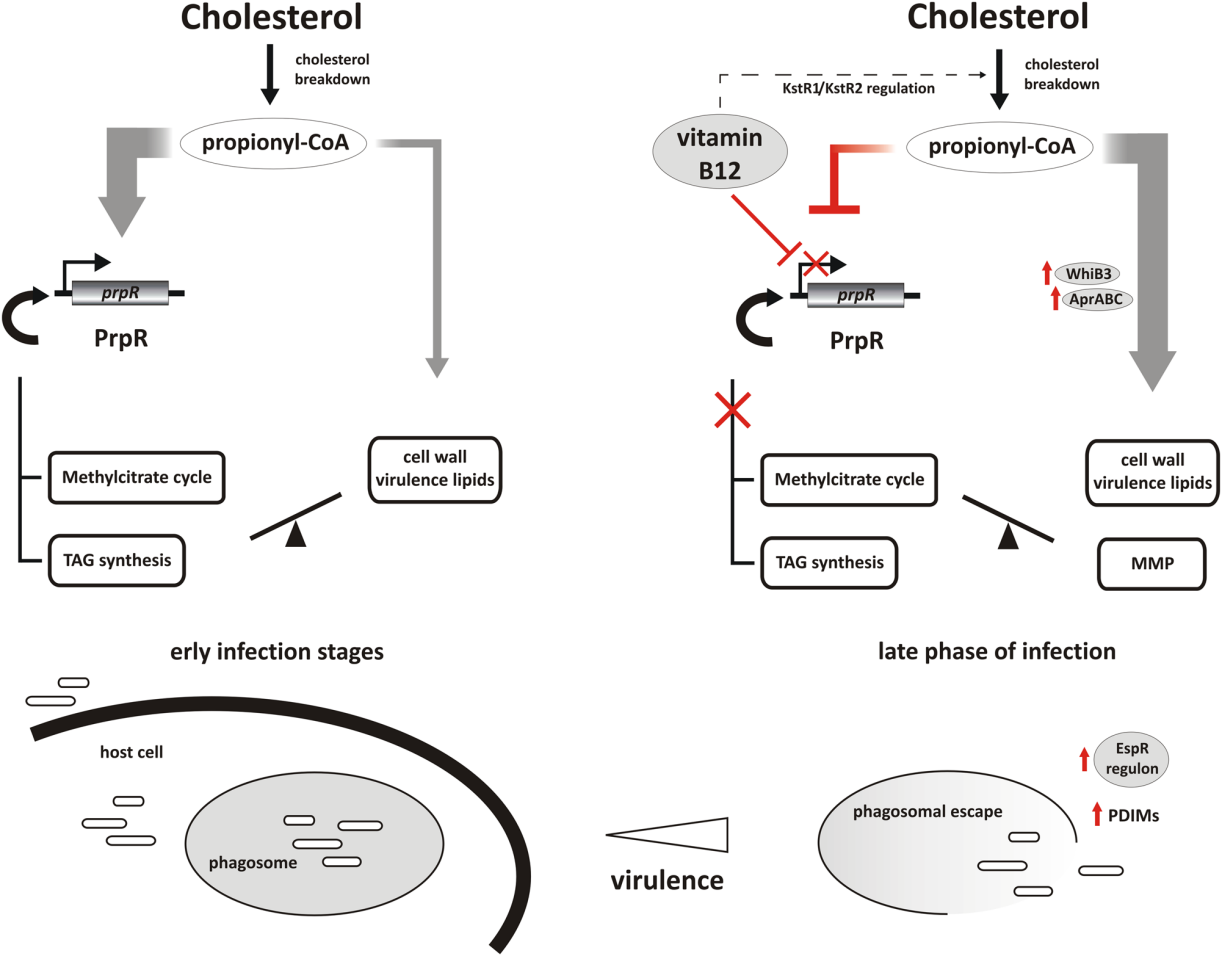
The role of vitamin B_12_ and PrpR joint action in the channeling of propionyl-CoA and virulent phenotype induction during *Mtb* infection. See the text for description.

In addition to the MCC, the generation of the TCA intermediate from propionyl-CoA can also be carried out by the methylmalonyl pathway (MMP), provided that vitamin B_12_, a cofactor of methylmalonyl-CoA mutase (as adenosylcobalamin), is supplied exogenously^51^. Despite being functional in *Mtb*, MMP did not respond transcriptionally to cholesterol even after MCC inhibition and/or vitamin B_12_ supplementation (Figs. 4, 5C). Therefore, the expression of MMP genes is rather continuous in *Mtb,* and the functionality of the whole pathway is regulated solely through B_12_ availability. It was previously suggested that in the presence of B_12_, both MCC and MMP cooperate to provide optimal growth on propionate^51^. However, our study revealed that providing *Mtb* with vitamin B_12_ not only renders MMP functional but also strongly decreases the expression of the *prpR* and *prpDC* regulon, thus inhibiting MCC. The observed effect of B_12_ is independent of the utilized carbon source (Figs. 4, 5B, D). RNA-Seq analysis of the *Mtb* wild-type strain growing in standard 7H9/10% OADC medium with or without vitamin B_12_ supplementation (Dataset S1) revealed that among the entire *Mtb* transcriptome, genes of the PrpR operon undergo the strongest repression on medium supplemented with B_12_ together with closely located *metE* encoding methionine synthase, whose regulation is a confirmed example of B_12_-sensing riboswitch in *Mtb*^52^ (Fig. 5D, Dataset S1). Whether MCC genes are regulated through the same mechanism requires further study. Previous studies demonstrated that an *Mtb* mutant with impaired B_12_ import reveals no impact on the early establishment of infection but a profound effect on the maintenance of chronic disease^53^. Therefore, both the B_12_-dependent and B_12_-independent pathways of cholesterol metabolite dissipation are essential for maintaining infection; however, we can speculate that they operate in a specific sequence imposed by the identified interaction between PrpR and B_12_ (Fig. 6). We propose that at the early infection stage, intensive cholesterol consumption highly increases the expression of PrpR. PrpR directs carbon flux through the MCC and GC and induces TAG biosynthesis. It is uncertain whether B_12_ is continuously available in *Mtb* cell during infection or becomes available at some distinct stage. Nevertheless, under an unclear stimulus, which may be a change in internal redox potential and/or pH^54^, B_12_ inhibits the expression of PrpR and pathways controlled by this regulator, and this change directs most of the propionate flux to virulence lipid synthesis. In addition, B_12_-unlocked MMP allows for efficient conversion of some part of carboxylated propionate into succinate, thereby bypassing down-regulated MCC and the need for anaplerosis *via* GC (Fig. 6). Moreover, B_12_ strongly up-regulated the AprA regulator, inducer of PDIM biosynthesis (Dataset S1, Fig. 6). Induction of the *aprABC* locus, a modulator of macrophage phagosome adaptation, has been described to date as pH-driven only^55^. An increase in the amount of cell wall virulence lipids at the late phase of infection facilitates phagosomal escape and host cell exit, allowing *Mtb* dissemination^56^. Indeed, the gene pool that significantly changed its expression in response to B_12_ also includes members of the EspR regulon (Dataset S2, Figs. 5D, 6) that are directly involved in phagosomal escape and work synergistically with PDIM to help *M. tuberculosis* escape the host cell^56^. Interestingly, in addition to affecting the route of intermediary metabolite amelioration, B_12_ may also control their accumulation rate by down-regulating the expression of KstR2 and KstR1 genes (cholesterol metabolism) and by regulation of fatty acid metabolism (MabR, FasR regulon genes) (Fig. 6, Dataset S2). Overall, we noticed a B_12_-induced transcriptional change in many *Mtb* regulons involved in survival under various stresses, cell wall integrity, antibiotic resistance and tolerance to toxic compounds, cell division or regulation of latency (Dataset S2). Therefore, vitamin B_12_ may not only be an enzyme cofactor but also an important regulator coordinating the shift in *Mtb* metabolism and virulence in response to the variable environment inside the host.

### Cholesterol induces DosR/S/T regulon

DosR regulates the expression of more than 50 genes involved in the transition of actively replicating *Mtb* to dormant bacilli upon gradual depletion of oxygen inside granuloma^57^ and resumption of growth upon return to normoxia^58^. Induction of *tgs-1*, a member of the DosR/S/T regulon, in cholesterol medium prompted us to analyze the cholesterol-induced response of the other Dos genes. Our study revealed that cholesterol in the *Mtb* growth environment is a potent inducer of the Dos response, even in actively replicating cells, much earlier than specific hypoxia symptoms. 36 of 51 Dos genes showed significantly up-regulated expression (Fig. S2). Importantly, *tgs-1* is the only Dos gene whose induction on cholesterol is inhibited in response to *prpR* deletion, thereby confirming that TAG synthesis is PrpR- rather than DosR-regulated on this carbon source. Although previous transcriptomic studies mention some Dos genes as induced during *Mtb* growth in the lipid environment^14,15^, the effect of cholesterol is the strongest reported so far.

### Cholesterol supports the development of Mtb drug tolerance and prevents the inhibition of own metabolism

**Figure 7:**
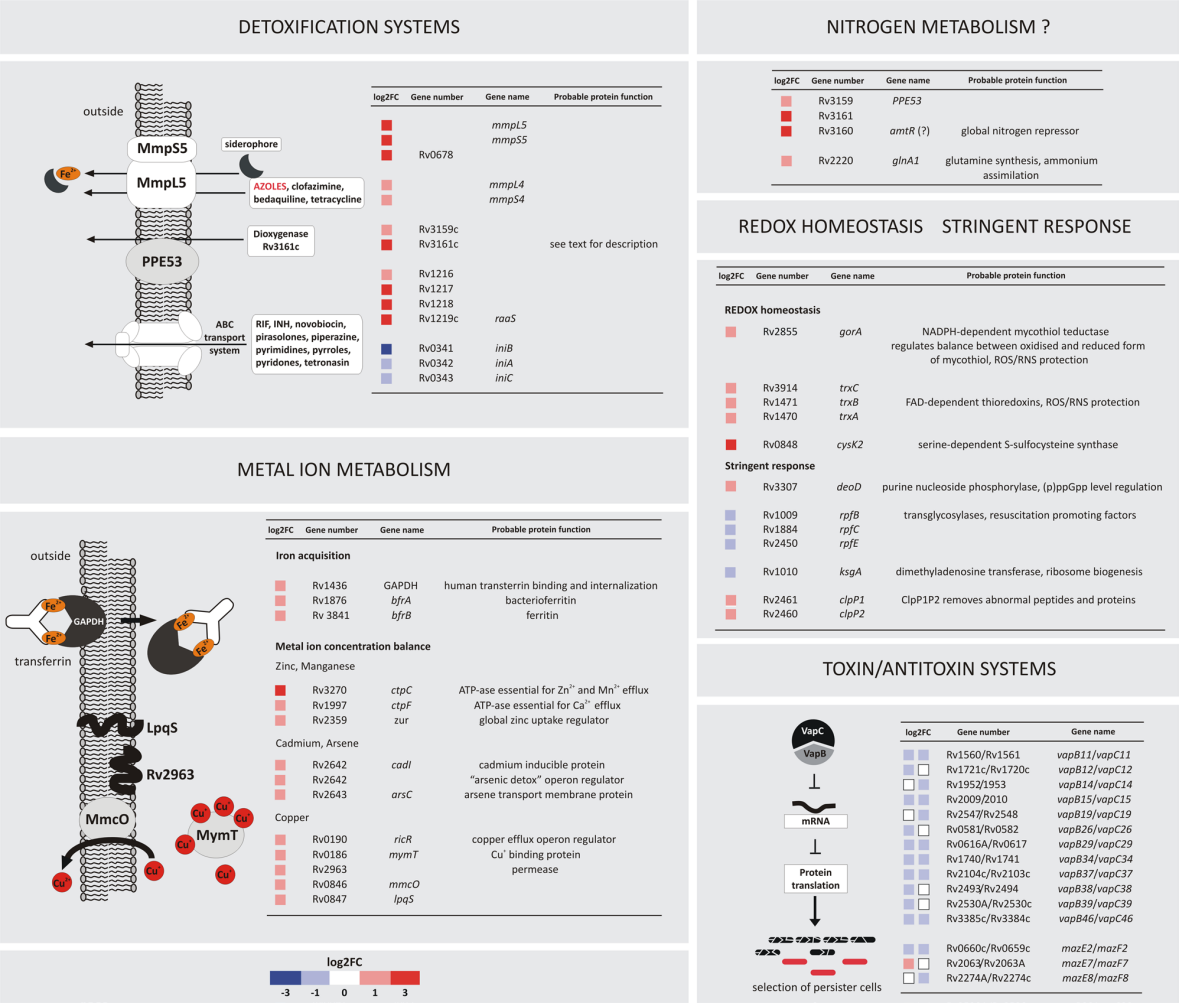
Examples of *Mtb* metabolic pathways that are modulated in a cholesterol-rich environment. Changes in the expression of the selected representative genes are shown. See the color key for log2FC values.

The majority of *Mtb* genes with the highest (≥ 4 log FC) change in expression on cholesterol encode efflux pumps conferring resistance to numerous antibiotics. Cholesterol greatly increased the expression of MmpS5/MmpL5 multidrug efflux pump components (Fig. 7). Together with *mmpS4*/*L4*, they are the only members of the *mmpS*/*L* family up-regulated on cholesterol and have been linked to the iron acquisition mechanism^59^. In addition to resistance to various antibiotics, MmpS5-L5 efflux determines *Mtb* resistance to azoles that target cholesterol metabolism by inhibiting cytochrome P450 (Cyp) monooxygenases^60,61^. Some *Mtb* Cyps, including Cyp125 and Cyp142, have already been demonstrated to bind azole drugs^62^. Up-regulation of MmpS5-L5 during growth on cholesterol may, therefore, secure continuity of cholesterol metabolism in two ways: by providing iron for many heme-containing catabolic enzymes and/or by effluxing heme-binding inhibitors. Cholesterol also up-regulates the *Rv1216c*-*Rv1218c* operon encoding the main ATP-dependent efflux pump conferring *Mtb* resistance to many chemically unrelated antibiotics^63^ (Fig. 7). The operon is up-regulated together with its regulatory protein RaaS (Rv1219c), which plays an important role in mycobacterial long-term survival *in vitro* and *in vivo*^64^. The activity of the mentioned efflux systems is strictly associated with the ability to survive within macrophages during latency. These systems were previously reported as co-induced in the presence of drugs or during growth in a mixture of various lipids^15,65^. However, as demonstrated here, the sole presence of cholesterol is a sufficient condition for such induction. Interestingly, cholesterol not only up-regulates *Mtb* efflux systems. The *iniBAC* operon, which encodes a pump-like efflux mechanism conferring tolerance to multiple anti-TB drugs^66^, was significantly down-regulated in this study (Fig. 7). *iniBAC* is highly induced in response to the inhibition of cell wall biosynthesis^67^; however, conditions that down-regulate *iniBAC* are, to date, largely unknown. Cholesterol affects not only *Mtb* drug efflux but also detoxification systems, such as the *Rv3161c-Rv3159c* gene cluster encoding a putative TetR regulator, probable dioxygenase, and outer membrane protein (Fig. 7). It is induced by various aromatic compounds, such as thioridazine and triclosan^68,69^. The operon was also reported to be induced in a lipid-rich environment^15^; however, again our results demonstrate that cholesterol is a sufficient inducer. *Rv3160*, the most cholesterol-up-regulated gene in the present study, encodes a protein controlling the whole set of metabolic events during active infection, dormancy, or resuscitation of dormant “nonculturable” *Mtb*^70–72^. Rv3160 is also considered a homolog of *C. glutamicum* AmtR, a global nitrogen metabolism regulator controlling ammonium uptake and assimilation^73^. Although this pathway in *Mtb* may be GlnR- rather than AmtR-regulated we have also observed, despite providing a nitrogen source in the culture media, cholesterol-dependent up-regulation of *glnA1* (Rv2220), which encodes glutamine synthetase essential for ammonium assimilation and an important determinant of *Mtb* pathogenesis^74^.

### Metal ion metabolism

*Mtb* has developed numerous mechanisms for maintaining cellular metal ion balance, thereby preventing intoxication inside the phagosome^75^. Here, we demonstrated that growth on cholesterol up-regulates the expression of many metal ion efflux/uptake systems and their regulators (Fig. 7). Many of them, such as the copper-responsive regulon RicR or “arsenic detox” operon, are essential for *Mtb* infection and *in vivo* growth, regulate drug susceptibility and are described as new virulence factors^76,77^. We have also noticed that despite providing iron in the culture medium, cholesterol specifically up-regulates genes of glyceraldehyde-3-phosphate dehydrogenase (GAPDH) (Rv1436) and iron storage protein ferritin (*bfrB*) (Rv3841), although their up-regulation has been previously only associated with iron depletion^78^. Both GAPDH and ferritin promote the most efficient and essential for virulence, siderophore-independent iron acquisition system that allows binding and internalization of the whole human transferrin^78^. Cholesterol-dependent GAPDH induction may thus secure iron for the sterol degradative pathway.

### Cholesterol modulates the expression of Mtb persistence-associated genes

We noticed a cholesterol-induced change in the expression of many genes seemingly unrelated in terms of supposed function. However, a closer look suggests their common contribution in providing a metabolic background for *Mtb* persistence and long-term dormancy. Cholesterol substantially up-regulates genes encoding mycothiol-dependent redox mechanism (Fig. 7), which is first-line defence against reactive oxygen and nitrogen species inside the host cell^79^. These genes determine entering persistence and are overexpressed in various dormancy models^80,81^. Importantly, the mycothiol switch controls *Mtb* cholesterol catabolism in response to the redox state inside the host macrophage^54^. Long-term survival of nonreplicating *Mtb* is ensured by the coordinated shutdown of active metabolism through the stringent response mediated by hyperphosphorylated guanosine ((p)ppGpp)^82^. Cholesterol induces the expression of purine nucleoside phosphorylase DeoD (Rv3307), which is thought to be involved in the Rel-dependent regulation of (p)ppGpp levels^83^ (Fig. 7). Its expression correlates with dormancy and is considered among the top 100% persistence targets (TBSGC, www.webtb.org). Strikingly, we observed also cholesterol-induced change in expression of genes encoding resuscitation-promoting factors (Rpfs) coordinating the transition between *Mtb* nonreplicating persistence and active growth^84^. *Mtb* grown in cholesterol down-regulates three (*rpfBCE*) of five Rpf genes as well as cotranscribed and coregulated *ksgA* (Rv1010)^85^. KsgA methyltransferase is involved in ribosome biogenesis; thus, its down-regulation affects translation and growth^86^. Intriguingly, one of the most represented groups of *Mtb* genes that changes expression in response to cholesterol is a toxin-antitoxin (TA) system. Among 15 TA pairs with altered expression, 13 are represented by the VapBC family. It is expected that in response to various stressors, VapC activity generates the stochastic expression of many proteins interfering with key cellular processes, ultimately leading to the emergence of persister cells^87,88^. Therefore, in this paper, we have demonstrated a link between cholesterol in the *Mtb* growth environment and a specific metabolic program that may facilitate entering dormancy, resumption of growth upon exposure to ambient O_2_ levels and selection of persister cells.

## Conclusions

Cholesterol is considered essential for *Mtb* to maintain a chronic infection; however, the molecular basis of this phenomenon is largely unknown. In this study, we delineated the transcriptomic landscape of tubercle bacilli that utilize cholesterol as the sole carbon source, which provided insights on the contribution of cholesterol to establishing persistent infection. The data allowed us to verify the list of cholesterol breakdown genes that are truly cholesterol-induced. We further proposed a new function of vitamin B_12_ in directing cholesterol metabolites which, together with analysis of B_12_-induced changes in *Mtb* transcriptome, is the first attempt to the identification of the role of cobalamin in mycobacterial virulence. Apparently many changes in *Mtb* transcriptome that were previously ascribed to the lipid-rich environment are in fact induced by cholesterol. We demonstrated that the essential role of cholesterol in *Mtb* intracellular survival is not only coupled to central carbon metabolism and energy production but also to the ability to induce a transcriptomic program that promotes persistence. Therefore, cholesterol may facilitate the development of *Mtb* tolerance to unfavorable host cell environment far before specific stress-inducing phagosomal signals occur. The direct mechanism of this transcriptome remodeling program demands further study.

## Methods

### Bacterial strains and culture conditions

The *Mycobacterium tuberculosis* H37Rv wild-type strain and derived mutants (described below) were maintained on Middlebrook 7H10 agar and 7H9 broth (Becton-Dickinson) supplemented with 10% OADC enrichment (Becton-Dickinson). For RNA sequencing, the *Mtb* wild-type and ∆*prpR* mutant were grown on defined carbon sources in minimal medium (0.5 g/liter asparagine, 1 g/liter KH_2_PO_4_, 2.5 g/liter Na_2_HPO_4_, 50 mg/liter ferric ammonium citrate, 0.5 g/liter MgSO_4_∙7H_2_O, 0.5 mg/liter CaCl_2_, and 0.1 mg/liter ZnSO_4_) containing either 0.1% glycerol (vol/vol) or 0.01% cholesterol (wt/vol)^7^. RNA sequencing data resulting from a comparison of the expression profile of glycerol vs cholesterol were also related to the results obtained for bacteria growing in standard 7H9/10% OADC medium, which is referred to as a “rich medium”. For the study on the influence of vitamin B_12_ on the *Mtb* transcriptome, bacteria were grown in 7H9/10% OADC medium with or without vitamin B_12_ (Cyanocob(III)alamin) (Sigma Aldrich) supplementation (10 μg/ml). Each bacterial culture was set in triplicate and grown to an optical density of OD_600_ 0.6. Then, 100 ml of each culture was spun down and subjected directly to total RNA isolation. Minimal medium supplemented with 0.01% cholesterol (wt/vol) was also used in cholesterol uptake and biotransformation experiments.

### Library preparation and sequencing

For total RNA sequencing, RNA was isolated using TRIzol LS reagent (Invitrogen) as described previously^89^. Cells were lysed by bead beating with the MP FastPrep system (MP Biomedicals) using lysing matrix B. DNA contamination of the RNA samples was removed by DNase I turbo (Invitrogen) digestion according to the manufacturer’s protocol. The quantity and integrity of the DNase I-treated RNA was assessed using an Agilent 2100 BioAnalyzer according to the manufacturer's instructions (Agilent RNA 6000 Nano Kit). The Illumina-compatible RNA/cDNA sequencing libraries were prepared essentially as described earlier^90^. For total RNA sequencing, RNA samples were purified using AMPure XP magnetic beads (Becton Dickinson) with a 2.2:1 bead-to-RNA ratio. Two micrograms of the purified total RNA was used per library. The ribosomal RNA was removed with the Ribo-Zero Bacteria rRNA Removal Kit (Illumina) according to the manufacturer’s protocol. Ribodepleted RNA was used to generate cDNA libraries with the KAPA Stranded RNA-Seq kit (Roche) according to the detailed instructions provided by the manufacturer. Thirteen amplification cycles were performed on the initial cDNA libraries to obtain the final sequencing libraries. The Illumina True Seq v2 indexing system was used as a barcoding system for multiplex sequencing. The quality and quantity of the resulting libraries were assessed with the help of an Agilent 2100 BioAnalyzer fitted with a DNA 1000 chip. Before assembling the sequencing run, the libraries were additionally quantified by qPCR with the NEBNext Library Quant Kit for Illumina (New England Biolabs). The NextSeq500 System (Illumina) with the NextSeq 500/550 Mid Output v2 sequencing kit (150 cycles) (Illumina) was used to sequence RNA-Seq libraries, ensuring between 5 and 10 million paired-end reads per sample.

### RNA-Seq data analysis

Demultiplexed high-throughput RNA sequencing results were processed with a series of software and bioinformatic scripts as previously described^90^. Briefly, after the initial removal of sequencing adapters with Cutadapt v. 1.9.1^91^, the sequencing reads were quality trimmed with the windowed adaptive trimming tool Sickle, which allowed for a minimum quality of 30% and a minimal read length of 20 bp. Reads of sufficient quality and length were next aligned to the *M. tuberculosis* H37Rv genome (NC_018143.2) using the Bowtie2 short read aligner^92^. SAMtools^93^ software suite was used for aligned data handling, conversion, and indexing. Gene-mapped reads or read pairs were counted according to appropriate genomic features (coding DNA sequences - CDSs) with the help of the HTSeq 0.6.1 python module^94^. Integrative Genomics Viewer (IGV) was used to visualize sequencing results in the genomic context^95^. The gene expression matrix file was generated by merging individual HTSeq output files, with the gene name treated as an index. The resulting file was submitted to the online Degust RNA-Seq analysis platform, which was used to estimate the differential expression. Default parameters were used during the analysis, with voom/limma algorithms set for differential gene expression estimation (http://degust.erc.monash.edu/)^96^. The statistical analysis of differential gene expression (DGE) was performed by Degust and is provided as the false discovery rate (FDR) value. To identify significant changes in gene expression, genes with an FDR of <0.05 and a log_2_ fold change greater than an absolute value of 1 (changing two times or more) were considered differentially expressed in the current study. The whole procedure from bacterial culture to cDNA library sequencing was performed in three independent replicates for each strain/condition studied, and the obtained RNA-Seq data were averaged during Degust analysis. The RNA-seq related data have been deposited in NCBI's Gene Expression Omnibus (GEO) database and are accessible through GEO Series accession number GSE175812 (https://www.ncbi.nlm.nih.gov/geo/query/acc.cgi?acc=GSE175812).

### qRT-PCR validation of selected genes

To validate the RNA-Seq results, a qRT-PCR analysis was performed for selected genes. SuperScript III First-Strand Synthesis SuperMix (Invitrogen) was used for reverse transcription of relevant RNA samples according to the manufacturer’s instructions. Subsequently, 1 μl of cDNA (equivalent to 50 ng of RNA) was used in the qRT-PCR experiment. qRT-PCR was performed using Maxima SYBR Green qPCR master mix (Thermo Scientific) and a 7900HT real-time PCR system (Applied Biosystems). To verify the specificity and identity of the PCR products generated, a melting curve analysis was performed at the end of each PCR.

### Constructions of gene replacement vectors

Standard molecular biology protocols were used for all cloning procedures^97^. All PCR products were obtained using thermostable AccuPrime *Pfx* DNA polymerase (Invitrogen). They were initially cloned into a pJET1.2/blunt vector (Thermo Scientific), followed by sequencing and digestion with the appropriate restriction enzymes, and then they were cloned into the final vectors. To facilitate subcloning, certain restriction enzyme recognition sites were incorporated into the primer sequences (see Table S1). To create an unmarked deletion of the *cyp125*, *cyp142*, or multigenic regions, *supA*-*supB* (*yrbE4A* – *yrbE4B*), *mce4A*-*mce4F,* and *fadD19*-*echA19*, suicidal recombination delivery vectors based on p2NIL^98^ were constructed (see Table S2). In the case of *cyp125* and *cyp142,* each vector carried the fragment upstream of the gene together with the 5′ end of the gene (GR1-GR2) cloned next to the 3′ end of the gene and its downstream fragment (GR3-GR4) cloned into p2NIL to generate p2N*cyp125*_*Tb*_ and p2N*cyp142*_*Tb*_ vectors, respectively. The GR1-GR2 and GR3-GR4 fragment lengths as well as the PCR primers used for their amplification on M*. tuberculosis* chromosomal DNA are listed in Table S1 and Table S2. For the deletion of the two-gene *supA*-*supB* region, the 5′ end of *supA* and the fragment upstream of a gene (GR1-GR2) were cloned next to the 3′ end of *supB* and its downstream fragment (GR3-GR4) to generate a p2N*sup*_*Tb*_ vector. For the deletion of the six-gene *mce4A*-*mce4F* region, the 5′ end of a gene together with the upstream sequence (GR1-GR2) was cloned next to the 3′ end of *mce4F* together with the sequence downstream of a gene (GR3-GR4) to generate the p2N*mce4*_*Tb*_ vector. For simultaneous deletion of the *fadD19* and *echA19* genes, the suicidal vector carried the fragment downstream of the *fadD19* together with the 3′ end of the gene (GR1-GR2) was cloned next to the 3′ end of the *echA19* and its downstream fragment (GR3-GR4) to generate the p2N*fadech*_*Tb*_ vector. All GR1-GR2 and GR3-GR4 fragments in the gene replacement vectors were ligated out of frame, resulting in the expression of nonfunctional proteins.

### Disruption of genes by homologous recombination

The plasmid DNA of p2N*cyp125*_*Tb*_, p2N*cyp142*_*Tb*_, p2N*sup*_*Tb*_, p2N*mce4*_*Tb*_ and p2N*fadech*_*Tb*_ suicide delivery vectors was UV-treated and electroporated into wild-type *M. tuberculosis* competent cells. The two-step homologous recombination protocol of Parish and Stoker^98^ was used to introduce unmarked deletions into the above-described genes or gene regions. The genotypes of selected double-crossover mutants ∆*cyp125*, ∆*cyp142*, ∆*supAB*, ∆*mce4AF*, and ∆*fadD19*∆*echA19* were confirmed by PCR and Southern blot hybridization on a chromosomal DNA template digested with BglII, ClaI, BamHI, PstI, and ClaI (Fig. S1). Hybridization probes were generated by PCR on the appropriate gene replacement vector DNA as the template. Probe labeling, hybridization, and signal detection were performed using the AlkPhos Direct labeling and detection system (GE Healthcare) according to the manufacturer’s instructions. For the construction of the ∆*supAB*∆*kstD*, ∆*mce4AF*∆*kstD*, and ∆*fadD19*∆*echA19*∆*kstD* mutants, ∆*supAB*, ∆*mce4AF*, ∆*fadD19*∆*echA19* strains were used as the recipients of a *kstD* gene replacement vector. Disruption of *kstD* by homologous recombination and genotype verification were performed as described elsewhere^3^. The construction of the *M. tuberculosis* ∆*prpR* mutant was described previously^41^.

### Cholesterol biotransformation

Wild-type *M. tuberculosis* and the constructed mutant strains were cultured in minimal medium supplemented with 0.01% cholesterol^7^. To follow the process of cholesterol biotransformation and detect intermediates, five-milliliter culture samples were withdrawn from the culture at 24 h intervals and extracted three times with an equal volume of chloroform. The extracts were dried under vacuum, the residue was dissolved in 0.5 ml of acetone, and the isolated steroids were analyzed by gas chromatography as previously described^99^. To quantify the cholesterol and 9-hydroxy-4-androstene-3,17-dione (9OHAD), equal amounts of the internal standard 4-androstene-3,11,17-trione (Sigma) were added to each sample.

## Supplementary information

Supplementary Information 1.

Supplementary Information 2.

Supplementary Information 3.
