# Supplementary material for: Cholesterol-dependent transcriptome remodeling reveals new insight into the contribution of cholesterol to Mycobacterium tuberculosis pathogenesis
Source: Sci Rep. 2021 Jun 11;11:12396. doi: 10.1038/s41598-021-91812-0 (PMC8196197; doi:10.1038/s41598-021-91812-0)
Supplement: Supplementary file 3 — Supplementary Information 3. [file 41598_2021_91812_MOESM3_ESM.pdf]

Cholesterol-dependent transcriptome remodeling reveals new insight into the contribution of cholesterol to *Mycobacterium tuberculosis* pathogenesis

Jakub Pawełczyk, Anna Brzostek, Alina Minias, Przemysław Płociński, Anna Rumijowska-Galewicz, Dominik Strapagiel, Jolanta Zakrzewska-Czerwińska, Jarosław Dziadek

Supplementary Information:

Supplementary Figure S1. - Southern blot confirmation of the *Mtb* knockout mutants genotype.

Original images for Figure S1. - Southern blot photographic films that were used to crop the final images for preparing the Supplementary Figure S1. Cropped areas are marked with white frames. The blots were not cut prior to hybridisation. Cropping helped only to present the data from lines that were not adjacent to each other and to minimize the size of the whole figure.

Supplementary Figure S2. - *DosR/S/T* regulon genes upregulated in the transcriptome of *Mtb* growing on cholesterol as the sole carbon source. See the color key for log2FC values.

Supplementary Dataset S1. - see the Excel file

Supplementary Dataset S2. - see the Excel file

Supplementary Table S1. - Oligonucleotides used for PCR amplification in this study.

Supplementary Table S2. - Plasmid vectors and bacterial strains used in this study.

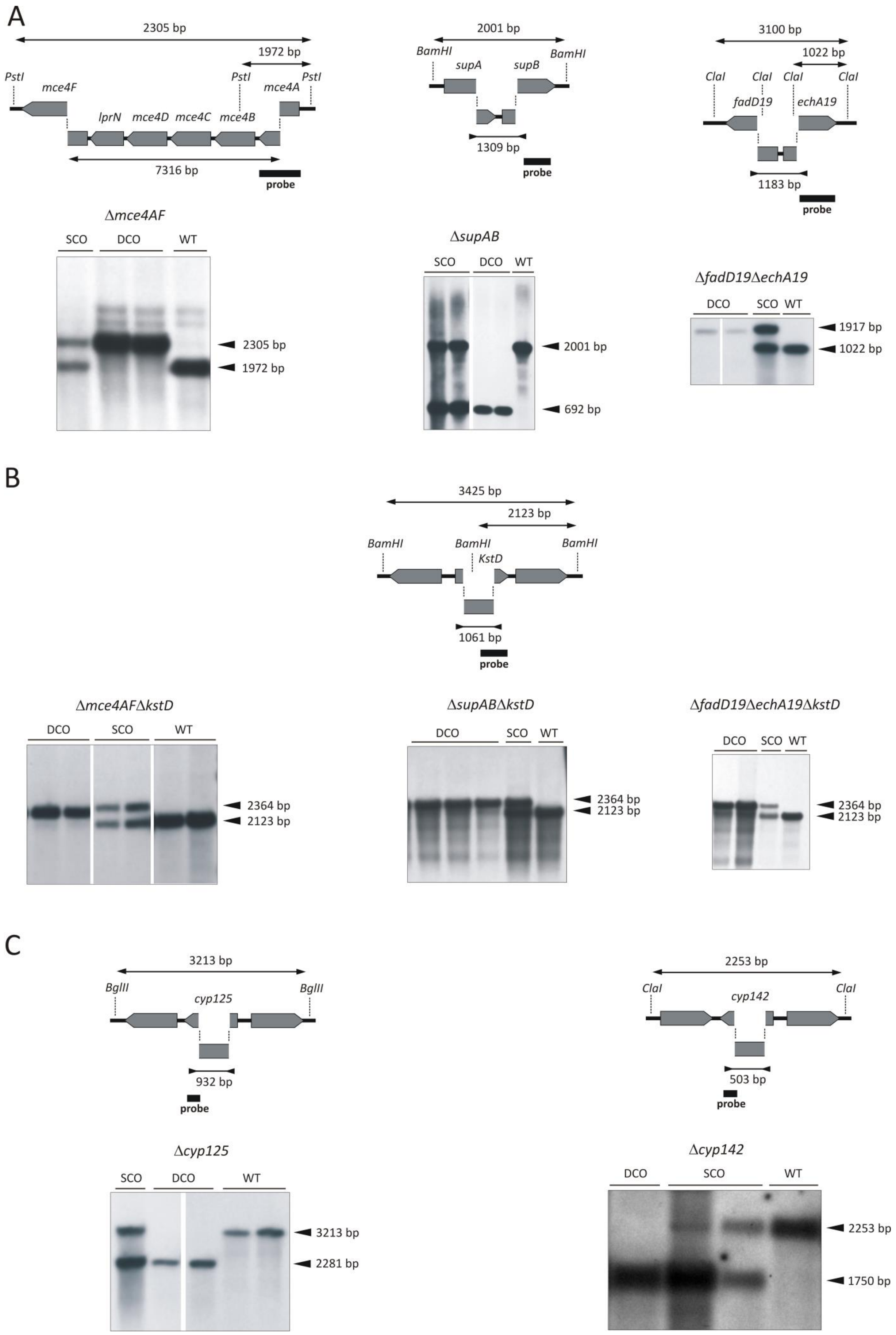

Supplementary Figure S1. Southern blot confirmation of the *Mtb* knockout mutants genotype. Diagrams show the size of deleted regions and restriction fragments after digestion with appropriate enzymes (PstI, BamHI, ClaI, BglII). The site of DNA probe hybridization is presented as a black bar. The size of the

restriction fragments hybridized to a DNA probe is marked next to the blots. Lines represent genomic DNA of WT – *Mtb* wild-type strain, SCO – single-crossover mutant carrying both wild-type and mutated copy of a gene, DCO – double-crossover mutant carrying a mutated copy of a gene. **A.** Southern blot confirmation of the Δ*mce4AF*, Δ*supAB* and Δ*fadD19*Δ*echA19* mutants genotype. **B.** Southern blot confirmation of the *kstD* gene deletion in Δ*mce4AF*Δ*kstD*, Δ*supAB*Δ*kstD* and Δ*fadD19*Δ*echA19*Δ*kstD* mutants. **C.** Southern blot confirmation of the Δ*cyp125* and Δ*cyp142* mutants genotype.

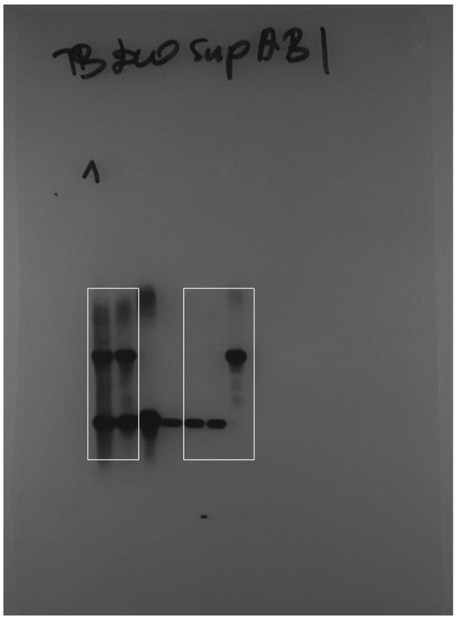

Refers to **Supplementary Figure S1 A (center)** – *supAB* mutant

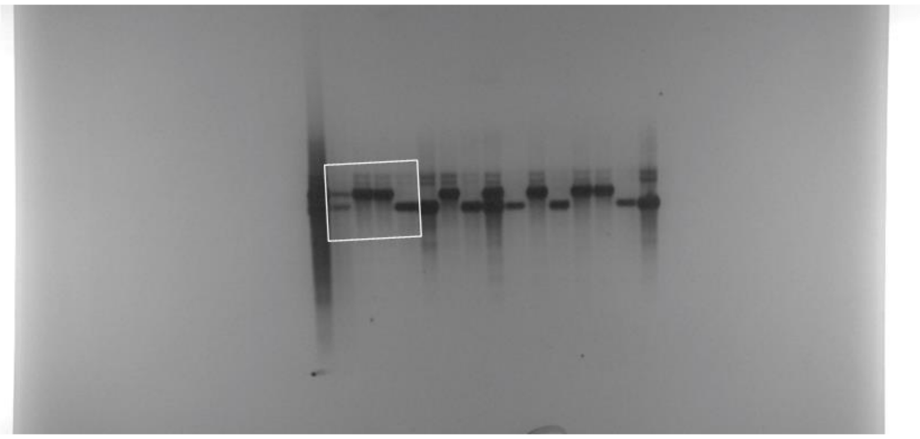

Refers to **Supplementary Figure S1 A (left)** – *mce4AF* mutant

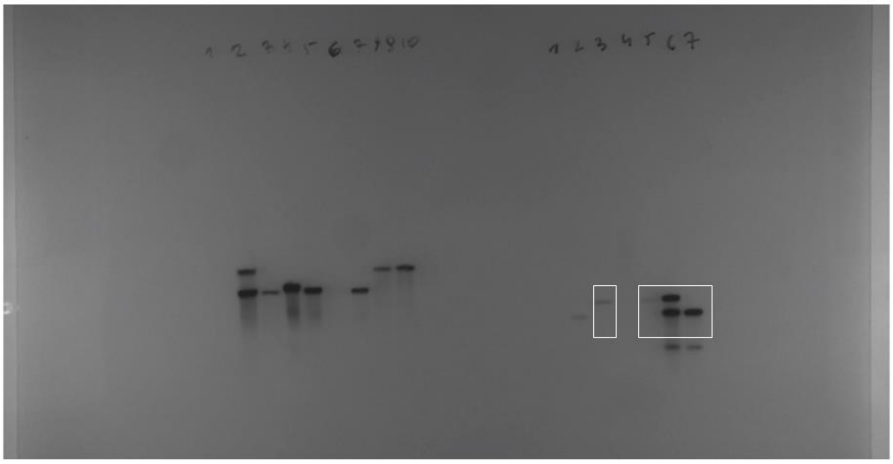

Refers to **Supplementary Figure S1 A (right)** – *fadD19echA19* mutant

Refers to **Supplementary Figure S1 B (left)** – *mce4AFkstD* mutant

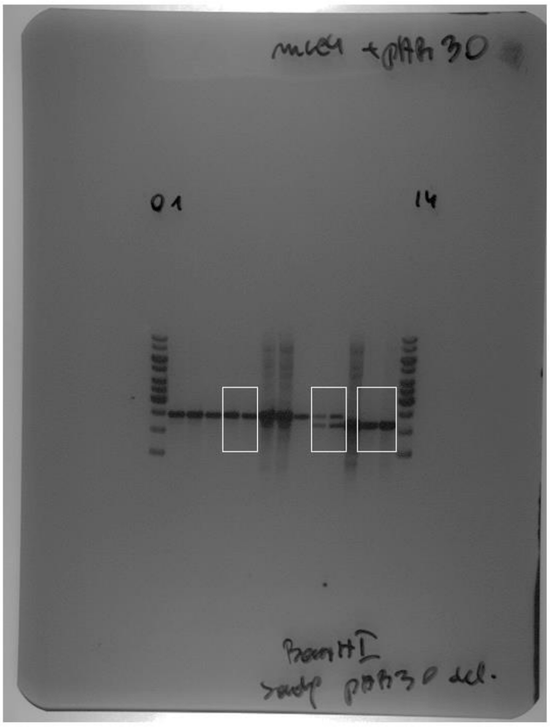

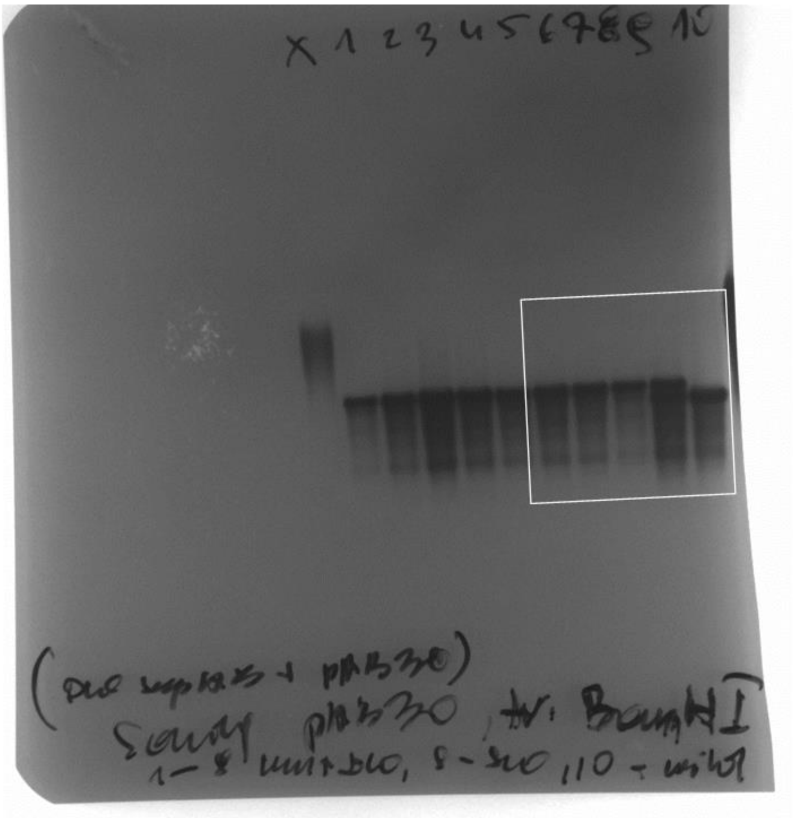

Refers to **Supplementary Figure S1 B (center)** – *supABkstD* mutant

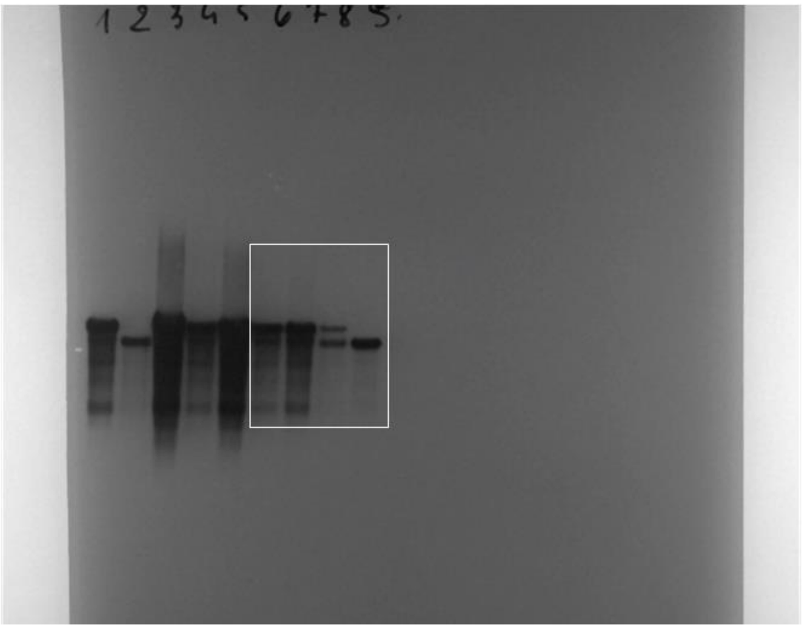

Refers to **Supplementary Figure S1 B (right)** – *fadD19echA19kstD* mutant

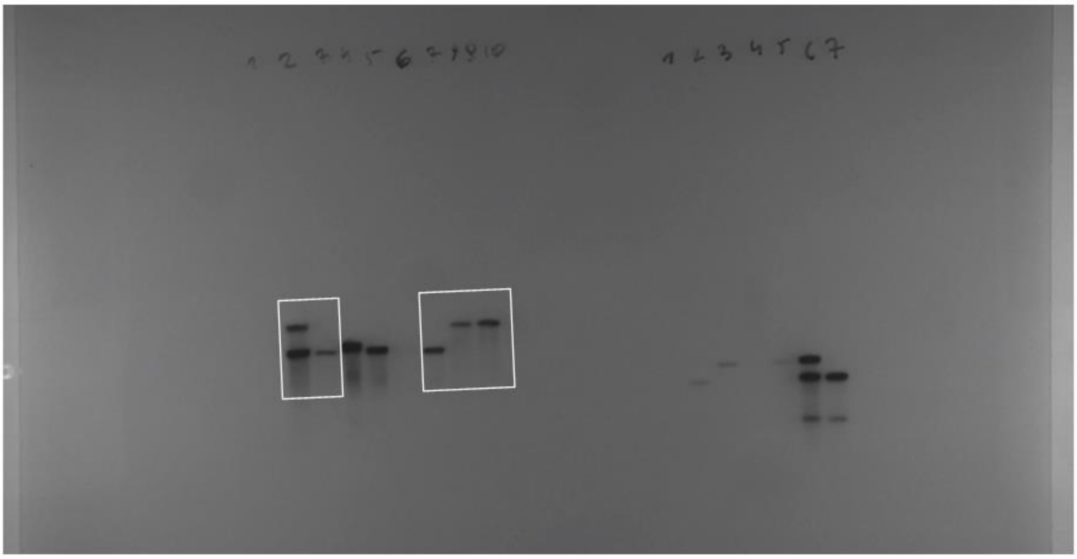

Refers to **Supplementary Figure S1 C (left)** – *cyp125* mutant

Refers to **Supplementary Figure S1 C (right)** – *cyp142* mutant

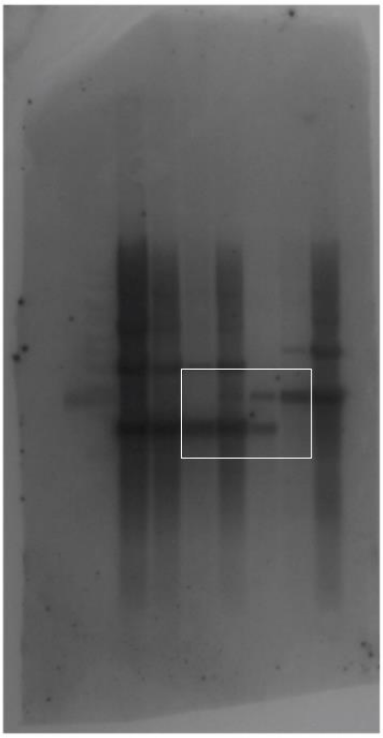

Original images for Figure S1. - Southern blot photographic films that were used to crop the final images for preparing the Supplementary Figure S1. Cropped areas are marked with white frames. The blots were not cut prior to hybridisation. Cropping helped only to present the data from lines that were not adjacent to each other and to minimize the size of the whole figure.

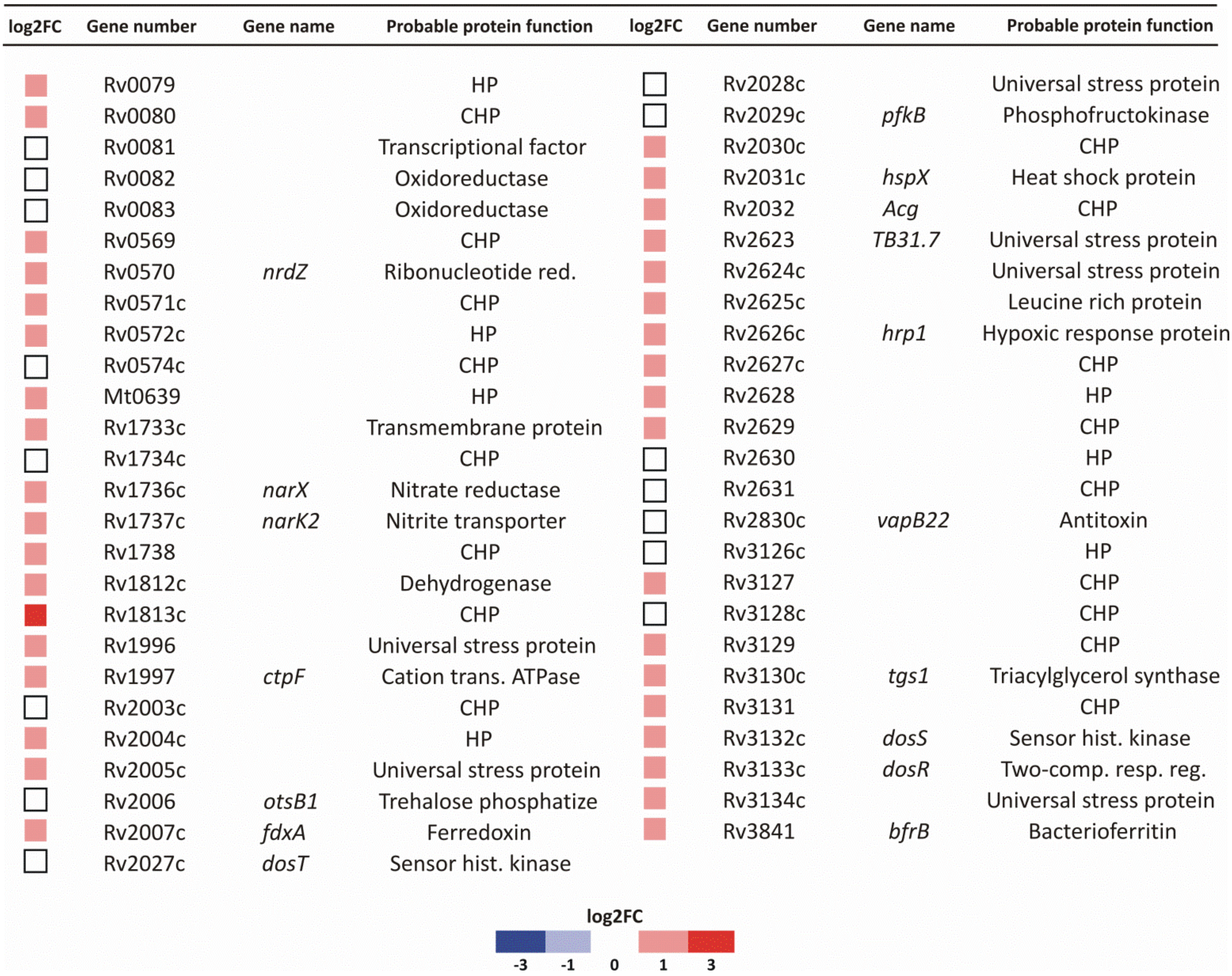

Supplementary Figure S2. ***DosR/S/T*** **regulon genes upregulated in the transcriptome of** ***Mtb*** **growing on cholesterol as the sole carbon source. See the color key for log2FC values.**

Primer	Sequence 5' → 3'	Description
**Construction of gene replacement vectors**		
**Rvmce-GR1**<br>**Rvmce-GR2**	F: GCTGCAGTACGGACCGGCTGCAGAACTTCAG<br>R: CAAGCTTCGGTCCGGCCTACACCATCTATGA	3' fragment of *mce4F* gene (145 bp) together with downstream sequence (1504 bp)
**Rvmce-GR3**<br>**Rvmce-GR4**	F: CAAGCTTGGATCCCACCATCAGCCCGG<br>R: CGGTACCGCAACGTGCGGGCCCTAAGC	5' fragment of *mce4A* gene (75 bp) together with upstream sequence (1734 bp)
**RvsupAB-GR1**<br>**RvsupAB-GR2**	F: AACTGCAGACCGTTTCCTCGGATGTAGCAGG<br>R: GCAAGCTTCATTTCGAAGAACCCGCCGAC	5' fragment of *supA* (*yrbE4A*) gene (54 bp) together with upstream sequence (1473 bp)
**RvsupAB-GR3**<br>**RvsupAB-GR4**	F: GCAAGCTTTTCGTCAATGGGCAGTCCGC<br>R: GGGGTACCCGTCTGCTTGGTCGGGATATCG	3' fragment of *supB* (*yrbE4B*) gene (279 bp) together with downstream sequence (1078 bp)
**Rvcyp142-GR1**<br>**Rvcyp142-GR2**	F: CAGTACTGTCGTGGAGGCAGGGATGAAGC<br>R: CAAGCTTGTTGGTTGGCCCGCATCCAC	5' fragment of *cyp142* gene (94 bp) together with upstream sequence (1850 bp)

**Rvcyp142-GR3**<br>**Rvcyp142-GR4**	F: CAAGCTTGACCTGGTCAGCGTGCTGGTGA<br>R: CGCGGCCGCAGGCGCTGACCAAGGCTCTAGA	3’ fragment of *cyp142* gene (600 bp) together with downstream sequence (1214 bp)
**Rvcyp125-GR1**<br>**Rvcyp125-GR2**	F: GTTGAGGTACCACATCGGGTTC<br>R: GCAAGCTTAGCCGTTCGGCGTAGATTG	5’ fragment of *cyp125* gene (113 bp) together with upstream sequence (1671 bp)
**Rvcyp125-GR3**<br>**Rvcyp125-GR4**	F: GCAAGCTTAGGTTTTCCAGGATCCGTTCAC<br>R: CTTCTTCTTCTGCAGCTCGCTG	3’ fragment of *cyp125* gene (257 bp) together with downstream sequence (1330 bp)
**Rvfadech-GR1**<br>**Rvfadech-GR2**	F: AACTGCAGCTGAAGCACCACCTGCCACC<br>R: GCAAGCTTTGATCCGCTACCCGATCCC	3’ fragment of *fadD19* gene (915 bp) together with downstream sequence (562 bp)
**Rvfadech-GR3**<br>**Rvfadech-GR4**	F: GCAAGCTTCCGGTGAAAGTGCGAAGTTC<br>R: ATGAGTAGCAGACGCAGCCAC	3’ fragment of *echA19* gene (414 bp) together with downstream sequence (1490 bp)
	**qRT-PCR primers**	
**mce4A-s**<br>**mce4A-r**	F: ACCGATTTGGCGCCGAAGAT<br>R: AAGGGCGCCAAGGTCAAATACC	*mce4A* (Rv3499c) expression analysis
**supA-s**<br>**supA-r**	F: ACTTAGCGGCTTGGGCGACG<br>R: GGAGAAGGGCGCCAAGGTCA	*supA* (*yrbE4A*) (Rv3501c) expression analysis
**kstD-s**<br>**kstD-r**	F: ACGAGGTCCTCAAGCGCCGC<br>R: CGGAGCCTCGGGGTAGTAGTCGG	*kstD* (Rv3537) expression analysis
**fadE30-s**<br>**fadE30-r**	F: TCGCCGAAGTGGTTGACCTTGT<br>R: CGAATTCGCGGCACTTAAGGG	*fadE30* (Rv3560c) expression analysis
**prpR-s**<br>**prpR-r**	F: TCTTCGATCTGGGCGCCGCT<br>R: GCTGGCCAAGGCCCTGGACTT	*prpR* (Rv1129) expression analysis
**icl1-s**<br>**icl1-r**	F: GCACCTACTCCGCCGAGGACGT<br>R: CTGCCAGCCCGACAGGTAGATGG	*icl1* (Rv0467) expression analysis
**fadD26-s**<br>**fadD26-r**	F: GTTTTAGCGCCACAAGGACTGGAA<br>R: GGCTGGCTGCGTATTTCGTTACAT	*fadD26* (Rv2930) expression analysis
**papA5-s**<br>**papA5-r**	F: CTGCGCGGTGTTATCGATGTCG<br>R: GCAATAGGGACACGCTCTGGTCG	*papA5* (Rv2939) expression analysis
**mutA-s**<br>**mutA-r**	F: CGTGCATTCCGGCTGGAAGG<br>R: AGATACACCCCGGACAGCAGCG	*mutA* (Rv1492) expression analysis
**mutB-s**<br>**mutB-r**	F: GATGTATGTGAACCAGCCGTGGACC<br>R: TGGAATCGATTGCCACACCGG	*mutB* (Rv1493) expression analysis
**tgs1-s**<br>**tgs1-r**	F: GGCGATCAGCTCGAATAACTGGTCT<br>R: TTCTTATCGTCGCTCGCTCAACG	*tgs1* (Rv3130c) expression analysis
**prpD-s**<br>**prpD-r**	F: GCCTACAAGATCGCCCAGGTGG<br>R: ATACCTTCGCCCCGTGTCGC	*prpD* (Rv1130) expression analysis
**metE-s**<br>**metE-r**	F: CGTCGACGGCCTTGCTCAGC<br>R: GGTTTCCGACGGGCTGGACC	*metE* (Rv1133c) expression analysis
**espA-s**<br>**espA-r**	F: TGGCGCCCTCCAGGATGTC<br>R: GCAGCAGCGTTTCCGGGTG	*espA* (Rv3616c) expression analysis
**sigA-s**<br>**sigA-r**	F: CCGATGACGACGAGGAGATC<br>R: CGGAGGCCTTGTCCTTTTC	*sigA* (Rv2703) expression analysis

Supplementary Table S1. Oligonucleotides used for PCR amplification in this study. Underlined regions indicate the restriction sites used during the cloning steps (PstI, HindIII, KpnI, ScaI, NotI, BglII). In some cases, natural restriction sites (KpnI, PstI, BamHI) were used.

Plasmid	Description	Source or reference
	**Cloning vectors**	
**pJET1.2/blunt**	PCR product cloning vector, Amp^R	Thermo Scientific
**p2NIL**	Recombination vector, nonreplicating in mycobacteria, Kan^R	[1]
**pGOAL17**	Source of PacI marker cassette (*sucB*, *lacZ*), Amp^R	[1]
	**Vectors used for gene replacement**	
**p2N*mce4*$_{Tb}$**	p2NIL-based recombination vector carrying the 3’ end of *mce4F* and its downstream flanking sequence (GR1-GR2) (1649 bp) cloned next to the 5’ end of *mce4A* and its upstream flanking sequence (GR3-GR4) (1809 bp),	This study

	enriched with the PacI cassette from pGOAL17, Kan^R	
**p2N*sup*$_{Tb}$**	p2NIL-based recombination vector carrying the 5' end of *supA* and its upstream flanking sequence (GR1-GR2) (1527 bp) cloned next to the 3' end of *supB* and its downstream flanking sequence (GR3-GR4) (1357 bp), enriched with the PacI cassette from pGOAL17, Kan^R	This study
**p2N*fadech*$_{Tb}$**	p2NIL-based recombination vector carrying the 3' end of *fadD19* and its downstream flanking sequence (GR1-GR2) (1477 bp) cloned next to the 3' end of *echA19* and its downstream flanking sequence (GR3-GR4) 1904 bp), enriched with the PacI cassette from pGOAL17, Kan^R	This study
**p2N*cyp125*$_{Tb}$**	p2NIL-based recombination vector carrying the 5' end of *cyp125* and its upstream flanking sequence (GR1-GR2) (1784 bp) cloned next to the 3' end of *cyp125* and its downstream flanking sequence (GR3-GR4) (1587 bp), enriched with the PacI cassette from pGOAL17, Kan^R	This study
**p2N*cyp142*$_{Tb}$**	p2NIL-based recombination vector carrying the 5' end of *cyp142* and its upstream flanking sequence (GR1-GR2) (1944 bp) cloned next to the 3' end of *cyp142* and its downstream flanking sequence (GR3-GR4) (1814 bp), enriched with the PacI cassette from pGOAL17, Kan^R	This study
**Bacterial strains used in this study**		
***M. tuberculosis* H37Rv**	*Mycobacterium tuberculosis* wild-type strain	Lab stock
**Δ*mce4AF***	*Mtb* H37Rv-based knockout mutant carrying an unmarked deletion (7316 bp) encompassing six genes of *mce4* operon (*mce4A*, *mce4B*, *mce4C*, *mce4D*, *lprN* and *mce4F*)	This study
**Δ*mce4AF*Δ*kstD***	Δ*mce4AF*-derived mutant containing additional unmarked deletion (1061 bp) within *kstD* gene	This study
**Δ*supAB***	*Mtb* H37Rv-based knockout mutant carrying an unmarked deletion (1309 bp) encompassing the 3' end of *supA* and 5' end of *supB* gene	This study
**Δ*supAB*Δ*kstD***	Δ*supAB*-derived mutant containing additional unmarked deletion (1061 bp) within *kstD* gene	This study
**Δ*fadD19*Δ*echA19***	*Mtb* H37Rv-based knockout mutant carrying an unmarked deletion (1183 bp) encompassing the 5' end of *fadD19* end *echA19* gene	This study
**Δ*fadD19*Δ*echA19*Δ*kstD***	Δ*fadD19*Δ*echA19*-derived mutant containing additional unmarked deletion (1061 bp) within *kstD* gene	This study
**Δ*cyp125***	*Mtb* H37Rv-based knockout mutant carrying an unmarked deletion (932 bp) within *cyp125* gene	This study
**Δ*cyp142***	*Mtb* H37Rv-based knockout mutant carrying an unmarked deletion (503 bp) within *cyp142* gene	This study
**Δ*prpR***	*Mtb* H37Rv-based knockout mutant carrying an unmarked deletion (1051 bp) within *prpR* (Rv1129c) gene	[2]

Supplementary Table S2. Plasmid vectors and bacterial strains used in this study.

References

1. Parish, T., Stoker, N. G. Use of a flexible cassette method to generate a double unmarked Mycobacterium tuberculosis tlyA plcABC mutant by gene replacement. *Microbiology* **146**, 1969-75 (2000).

2. Masiewicz, P., Brzostek, A., Wolański, M., Dziadek, J., Zakrzewska-Czerwińska, J. A novel role of the PrpR as a transcription factor involved in the regulation of methylcitrate pathway in Mycobacterium tuberculosis. *PLoS One* **7**, e43651 (2012).
